# Metasurfaces with Multipolar Resonances and Enhanced Light–Matter Interaction

**DOI:** 10.3390/nano15070477

**Published:** 2025-03-21

**Authors:** Evan Modak Arup, Li Liu, Haben Mekonnen, Dominic Bosomtwi, Viktoriia E. Babicheva

**Affiliations:** 1 Department of Electrical and Computer Engineering, University of New Mexico, MSC01 1100, 1 University of New Mexico, Albuquerque, NM 87131, USA; evanmarup@unm.edu (E.M.A.);; 2 Sandia National Laboratories, 1515 Eubank SE, Albuquerque, NM 87123, USA; bosomtwi@unm.edu

**Keywords:** metasurfaces, multipolar resonances, bound states in the continuum, spontaneous emission, photodetectors, nonlinearity, Kerker effect

## Abstract

Metasurfaces, composed of engineered nanoantennas, enable unprecedented control over electromagnetic waves by leveraging multipolar resonances to tailor light–matter interactions. This review explores key physical mechanisms that govern their optical properties, including the role of multipolar resonances in shaping metasurface responses, the emergence of bound states in the continuum (BICs) that support high-quality factor modes, and the Purcell effect, which enhances spontaneous emission rates at the nanoscale. These effects collectively underpin the design of advanced photonic devices with tailored spectral, angular, and polarization-dependent properties. This review discusses recent advances in metasurfaces and applications based on them, highlighting research that employs full-wave numerical simulations, analytical and semi-analytic techniques, multipolar decomposition, nanofabrication, and experimental characterization to explore the interplay of multipolar resonances, bound and quasi-bound states, and enhanced light–matter interactions. A particular focus is given to metasurface-enhanced photodetectors, where structured nanoantennas improve light absorption, spectral selectivity, and quantum efficiency. By integrating metasurfaces with conventional photodetector architectures, it is possible to enhance responsivity, engineer photocarrier generation rates, and even enable functionalities such as polarization-sensitive detection. The interplay between multipolar resonances, BICs, and emission control mechanisms provides a unified framework for designing next-generation optoelectronic devices. This review consolidates recent progress in these areas, emphasizing the potential of metasurface-based approaches for high-performance sensing, imaging, and energy-harvesting applications.

## 1. Introduction

**Figure 1:**
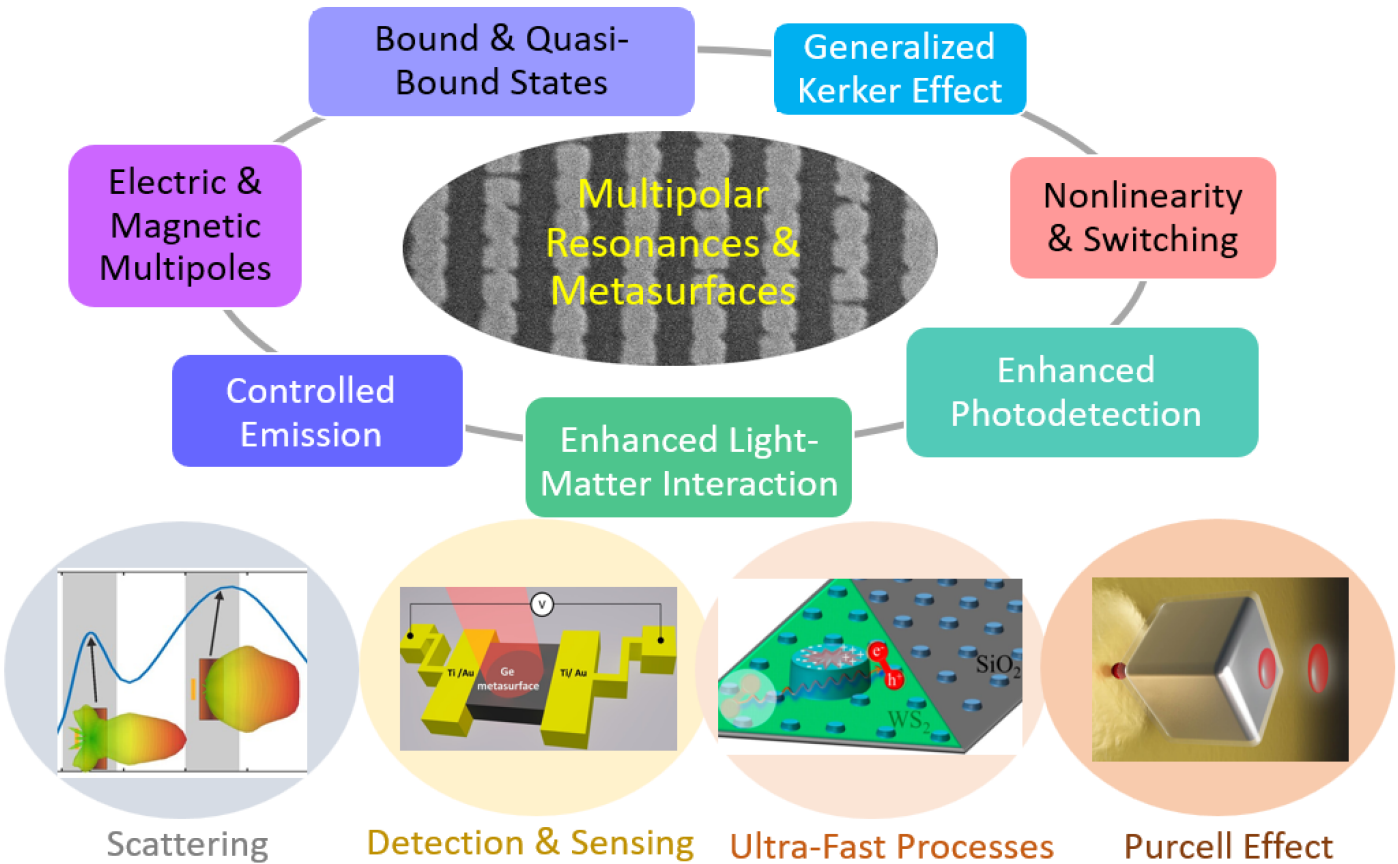
Multipolar resonances governing light scattering, the formation of bound states in the continuum enabling high confinement, and their impact on spontaneous emission and light–matter interaction. The integration of these concepts into metasurfaces enhances photodetector performance by improving absorption and quantum efficiency.

Recent rapid advances are driving the transition of metasurface research from fundamental science to practical technologies, marking the onset of the second metasurface revolution. Metasurfaces enable precise control over structural color by leveraging nanoantenna response, localized and delocalized resonances, and collective effects, and metasurfaces offer versatile and practical solutions for controlling light with unprecedented precision, enabling advancements across various technologies [1,2,3,4,5,6,7]. This allows for angle-independent, vivid, and tunable color generation in display and anti-counterfeiting technologies. In photodetection, metasurfaces enhance light absorption and spectral selectivity through engineered multipolar resonances, improving quantum efficiency and enabling compact, high-sensitivity detectors for imaging and optical communication. For light emission control, metasurfaces manipulate spontaneous emission via the Purcell effect and bound states in the continuum (BICs), facilitating efficient single-photon sources, directional emitters, and enhanced fluorescence for quantum optics and sensing applications (Figure 1). This shift is characterized by the integration of metasurfaces into real-world applications, leveraging their unparalleled ability to manipulate electromagnetic waves with subwavelength precision [8]. As fabrication techniques mature and new functionalities emerge, metasurfaces are poised to revolutionize fields such as imaging, sensing, communication, and computing.

Metasurfaces, composed of subwavelength-engineered nanoantennas, have revolutionized the control of light–matter interactions by tailoring electromagnetic responses at optical and infrared frequencies. Their ability to manipulate wavefronts, polarization, and spectral properties is derived from the underlying multipolar resonances, which define their scattering characteristics [9,10]. Understanding these resonances is essential for designing high-performance metasurfaces, as they govern fundamental optical phenomena such as BICs, the Purcell effect, and enhanced photodetection.

Despite significant advancements, metasurface research still faces several challenges that must be addressed to fully unlock their potential in practical applications. One major hurdle is the precise fabrication of complex nanostructures with subwavelength accuracy, as fabrication imperfections can degrade the designed optical responses. Additionally, losses in plasmonic metasurfaces and limited tunability in all-dielectric designs restrict efficiency and dynamic control, necessitating the development of low-loss materials and active tuning mechanisms. Scalability remains a concern, as large-area fabrication techniques often struggle to maintain uniform optical properties across extended surfaces. Furthermore, integrating metasurfaces with existing photonic and electronic platforms requires careful engineering to ensure compatibility and efficiency in hybrid systems. Addressing these challenges will be critical to advancing metasurface technologies in applications such as ultra-thin optical components, high-resolution imaging, and quantum photonics.

This review provides a comprehensive discussion of these key aspects, emphasizing the role of multipolar modes in shaping the optical response of nanoantenna-based metasurfaces. We begin by exploring multipolar resonances in nanoantennas and their role in tailoring metasurface responses, followed by an analysis of BICs, which enable high-quality-factor modes with suppressed radiation losses. The Purcell effect, crucial for enhancing spontaneous emission rates, is then discussed in the context of metasurfaces and their potential for controlling light emission at the nanoscale. Finally, we examine how metasurfaces improve photodetector performance by improving light absorption, quantum efficiency, and spectral selectivity. By unifying these topics, this review highlights how metasurfaces harness multipolar engineering to advance optical functionalities across diverse applications.

## 2. Multipolar Resonances

Interacting with nanoparticles, nanoantennas, and more complex structures with characteristic dimensions comparable to the wavelength, light waves generate spatial charge and current distributions, resulting in the emergence of resonance modes in such nanostructures [10,11]. Mie resonances, originally derived from the analysis of spherical particles, describe resonant scattering phenomena in all-dielectric nanoparticles and nanoantennas. These resonances, while applicable to a range of shapes, retain the same underlying nature as Mie’s analysis, involving the excitation of electric and magnetic multipolar modes that enhance light–matter interactions. Multipolar resonances are specific frequencies of the nanostructure at which higher-order multipolar moments, such as dipole, quadrupole, and octupole, resonate with incident electromagnetic waves. At these resonant frequencies, the system exhibits enhanced interactions with light, leading to strong scattering or absorption phenomena driven by these multipolar modes. These resonant modes, including electric, magnetic, and toroidal multipoles, are essential for a strong light–matter interaction on the nanoscale [12]. In plasmonic nanoantennas, electric multipolar resonances and their interference are prevalent with minimal magnetic responses, and the analysis can be expanded to higher-order electric multiples.

Mie resonances critically depend on distinguishing between electric and magnetic modes. While electric modes, also called electric multipoles, arise from oscillations of free charges and are characterized by strong electric fields at the nanoantenna surface, magnetic modes stem from displacement currents forming effective current loops that generate magnetic dipole moments. This distinction becomes particularly important in high-refractive-index dielectric nanoparticles, where magnetic Mie resonances can be excited with comparable strengths to their electric counterparts, enabling fascinating phenomena such as directional scattering, enhanced near-field distributions, and the ability to manipulate both the electric and magnetic components of light simultaneously. Understanding the different scaling behaviors, quality factors, and field distributions between these mode types allows us to design metaphotonic structures with precisely engineered optical responses, including applications in perfect reflection, zero forward scattering, and the creation of optical magnetism at frequencies where natural materials typically lack magnetic responses.

In moderate- and high-refractive-index nanostructures, light reflects from the boundaries multiple times with minimal loss, and, thus, the so-called Mie resonances emerge. In this case, light interacts with nanoparticles through the interplay of electric and magnetic fields with the particles’ electric and magnetic multipolar components [10]. Conventionally, the Kerker effect corresponds to the suppression of backward scattering when the permittivity of the spherical particle equals its permeability [13]. The Kerker effect refers to the phenomenon that occurs when the Kerker condition is satisfied, leading to the cancellation of specific scattering channels, such as forward or backward scattering. This condition, typically met in all-dielectric nanoantennas, results in enhanced light–matter interactions and unique directional scattering properties. The consideration can further be expanded using effective complex polarizabilities of nanoantennas having arbitrary shapes. In turn, the generalized Kerker effect involves angular scattering patterns and forward and backward directional radiation of the multipoles of types not only of electric dipoles (EDs) and magnetic dipoles (MDs) used in Kerker’s original work but also those of higher order [14]. Essentially associated with Mie resonances, the generalized Kerker effect utilizes multipolar excitations (analyzed through nanoparticle polarizability as core formalisms) and is vital for providing deeper insights into multipolar effects and light–matter interactions within nanoantennas, metastructures, and metasurfaces [10].

The generalized Kerker effect extends classical Kerker conditions by allowing precise control over the directional scattering of light using multipolar resonances in nanoantennas. Instead of just achieving zero backward or forward scattering with electric and magnetic dipoles, the generalized effect considers higher-order multiples, such as quadrupoles and octupoles, to tailor scattering at specific angles. This means that by carefully engineering the size, shape, and material properties of a nanoparticle or metasurface, light can be redirected in customized ways, enhancing applications like beam shaping, invisibility cloaking, and efficient light routing in photonic circuits. Unlike conventional scattering effects, which rely on simple interference between dipolar modes, the generalized Kerker effect provides a broader design space to achieve asymmetric and directional scattering across different wavelengths.

The excitation and interference of the resonant multipoles enable the effective control and manipulation of light in the optical range, as well as engineering the scattering response and tuning the radiation properties of nanophotonic devices [11,12]. Explicit analytical expressions for multipoles that result from the magnetization current density in nanophotonic structures can be used in conjunction with multipolar moments of the electric current density distribution [11]. An enclosed electromagnetic source has been shown to function as a pure-handed radiator whenever the multipolar moments with the spin current density and its transverse components are equal or oppositely relevant. Analytical expressions can also be used to explore the effects of enhanced currents generated in nanostructures in combination with other computational techniques, such as the discrete dipole approximation, as well as numerical Maxwell equations solutions.

Collective resonances in periodic arrays of nanoantennas arise from the coherent interaction of individual scatterers, leading to strong modifications in the optical response [15,16]. These resonances involve the excitation of various multipolar modes, including electric and magnetic dipoles, quadrupoles, and higher-order moments, whose interference governs the scattering properties [17]. The periodic arrangement facilitates lattice resonances, which can hybridize with intrinsic Mie resonances or plasmonic resonances of the nanoantennas, enhancing field confinement, the local density of states, and quality factors. This interplay can result in extraordinary near- and far-field enhancements, which enhance light–matter interactions, directional scattering, and spectral selectivity beyond what is achievable with isolated elements [18,19,20]. Periodicity plays a crucial role in lattice resonances by dictating the coupling between individual nanoantennas and shaping the overall optical response of a metasurface. When the periodicity of the array matches certain conditions, such as the Rayleigh anomaly, constructive interference between scattered waves leads to the formation of collective lattice resonances with enhanced quality factors. These resonances arise from the coherent interaction of multipolar modes across the array, resulting in strong near-field coupling and suppressed radiative losses. Adjusting the periodicity allows the fine-tuning of resonance wavelengths, enabling precise control over spectral features for applications in sensing, filtering, and nonlinear optics. By optimizing periodicity, metasurfaces can achieve high-*Q* resonances, directional scattering, and enhanced light–matter interactions, making them highly versatile for advanced photonic applications.

To ensure better reproducibility of metasurface simulations and experiments, key parameters such as material properties, structural dimensions, and excitation conditions must be explicitly defined. In numerical simulations, finite-difference time-domain and finite-element methods often model nanoantennas and their arrays with periodic boundary conditions, using optical constants extracted from experimental measurements [21]. Typical simulations consider wavelengths spanning the visible to infrared range, with resolutions fine enough to capture subwavelength features. Experimental studies rely on electron-beam or optical lithography or focused ion beam milling to fabricate metasurfaces with feature sizes on the order of tens to hundreds of nanometers, ensuring precise control over nanoresonator shape and geometry [22]. Optical characterization commonly involves dark-field or Fourier spectroscopy to measure scattering responses, while near-field scanning optical microscopy provides insight into localized field enhancements [23]. Specifying these parameters in detail allows for rigorous comparison between theoretical predictions and experimental realizations, facilitating advancements in metasurface design and application.

Nanoantennas based on metals and plasmonic nanostructures, in general, have been examined with the aim of achieving high directionality without backscattering [24] and the manipulation of light polarization [25]. Furthermore, the electrodynamics of conduction electrons upon exposure to an electromagnetic pulse is of particular interest [26]. Highly directional nanoantennas composed of a gold ring with an inner diameter of 80 nm, a thickness of 20 nm, and a height ranging from 40 to 150 nm have been proposed to generate balanced multipolar moments satisfying generalized Kerker conditions [24]. For example, the nanoring’s height of 150 nm results in ED mode scattering around 340 THz and electric quadrupole (EQ) mode scattering at roughly 400 THz, as well as backward scattering that is substantially restricted above 400 THz. The Kerker condition is satisfied when the ED and EQ components scatter light constructively at θ= 0°, causing an increase in the radiated electric field in the same direction.

The nonlinear optical response of a plasmonic nanowire approximated as a free-electron-metal wire with a radius of 3.5 nm has been explored by manipulating the spin and orbital angular momentum of incident light [26]. When excited with a Gaussian pulse of a circularly polarized time-varying electric field, confined multipolar modes and surface plasmon resonances can emerge along the circumference of the nanowire. This effect is related to multipolar order, the spectral components of induced charge density, and the near-field. Nonlinear near-field calculations with the time-dependent density functional theory have demonstrated the existence of higher-order multipolar moments of induced charge density. The *n*th-harmonic nonlinear response is generated by the induced field charge density, which also rotates along the transverse axis of the nanowire. All harmonics and time dependence are governed by *n* multipolar symmetry and spin–angular-momentum inversion is linked to system symmetry. The realization of nonlinear metasurfaces and high-harmonic-based on-chip components opens exciting opportunities for further fundamental analysis and practical applications.

Compact plasmonic nanoantennas with circular spin polarization and effectively coupled with semiconductor quantum dots (QDs) have been experimentally demonstrated [25]. Emitting as an ED, QDs overcome the constraint for the spin of the emitted photons on the nanoscale. Dominated by the multipolar radiation of the nanoantenna, the emission of chiral light with opposite handedness and a maximum ellipticity of ±0.5 has been observed in the QD–nanoantenna hybrid structure. Split-ring resonators composed of gold nanoantennas coupled with QDs generate spin-polarized photons near the wavelength of 800 nm, stunning from the combination of ED and EQ with two non-zero components. The nanoantenna as metasurface building blocks proves beneficial for multipolar designs, fluorophore detection, and quantum light sources because spin-controlled emission becomes increasingly crucial with the realization of QDs dispersed randomly among split-ring resonator arrays.

**Figure 2:**
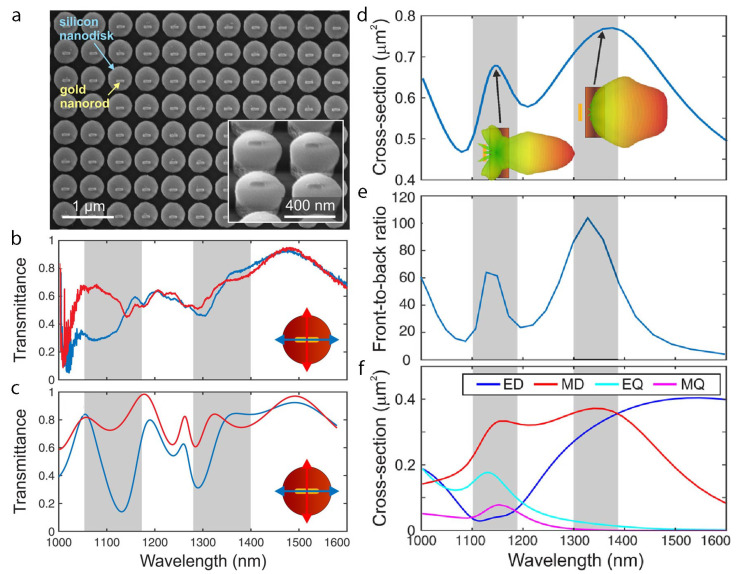
Optical characteristics of the gold–silicon hybrid metasurface. (**a**) Scanning electron microscopy image. Inset: Oblique view of enlarged nanoantennas. (**b**) Measured and (**c**) numerically simulated transmittance spectra of the metasurface. The red and blue curves demonstrate the transmittance under normally incident light with perpendicular and parallel polarizations with respect to the long axis of the gold nanorod, respectively. (**d**) Scattering cross-section obtained with simulations. (**e**) Front-to-back ratio of the light radiated by a hybrid nanostructure (with a unit cell of gold and silicon nanoantenna). (**f**) Multipolar decomposition of the incident and radiated optical field. The shaded area on panels (**d**) through (**f**) at the wavelengths of 1.1–1.2 μm and 1.3–1.4 μm shows two primary spectral regions where the multipolar components experience resonances. (**a**–**f**) Reproduced with permission from [22]. Copyright 2016 by American Chemical Society.

To realize the desired optical response, hybrid metal–dielectric metasurfaces can be engineered to possess high directionality and strong field enhancement based on plasmonic components in addition to high radiation efficiencies from dielectrics [22]. This hybrid metal–dielectric metasurface has been designed and realized with a two-step electron-beam lithography method that efficiently radiates light in a specific direction (Figure 2). A gold nanorod feed element and a silicon nanodisk director effectively excite multipolar resonances in the operating wavelength range of 1.1–1.4 μm. Individual metal–dielectric nanostructures harness the strengths of plasmonic gold nanorods and dielectric silicon nanodisks to induce mutual multipolar electric and magnetic responses. Within the specified spectral ranges of 1.1–1.2 μm and 1.3–1.4 μm, the metasurface has been characterized with incident perpendicular and parallel polarizations, yielding a maximum front-to-back ratio of 100 and substantially directed scattering patterns due to higher-order multipolar excitation in the silicon nanodisk. Furthermore, with a full 2π phase alteration with respect to incident light, the hybrid metasurface has the potential to become a practical component of optical and photonic applications.

All-dielectric metastructures, especially made of high-refractive-index materials, are considered to be very promising for nanoscale optical applications because they have low energy dissipation inside the nanoantenna material and substantial differences in refractive indices between the nanoantenna and free space. This enables strong light confinement and light–matter interactions, as well as enhanced Mie resonances compared to their plasmonic nanoantennas and metasurfaces [10]. Likewise, all-dielectric metasurfaces incorporate quasi-BIC and generate high-*Q* Fano resonances, with symmetrical meta-atoms and higher-order multipolar coupling by enabling strong field confinement, with the potential to improve applications of metastructures for sensing, nonlinear optics, and nanophotonics [27,28,29].

A comprehensive analysis has been conducted for metasurfaces that exhibit reflectivity as high as 95% [30]. At wavelengths of 1064 and 1550 nm, the disk-shaped silicon nanoantennas are positioned on top of the sapphire Al_2_O_3_ substrate to utilize its low refractive index, which in turn strengthens the metasurface reflectivity. The height of the silicon disks has been varied, resulting in tuning the resonances within the operating wavelength to adjust the ED and MD moments, as well as the resonant behavior of the entire metasurface. When the metamirror has been fabricated using a combination of dry etching and lift-off processes, the resonance of the MD moment is more pronounced than that of the ED moment. The Al_2_O_3_–air interface has been shown to contribute 15% additional reflections, which is considered negligible compared to the high reflection from the metasurface. The entire nanostructure yields more than 80% reflection, and the metamirrors are promising for next-era gravitational wave detection.

**Figure 3:**
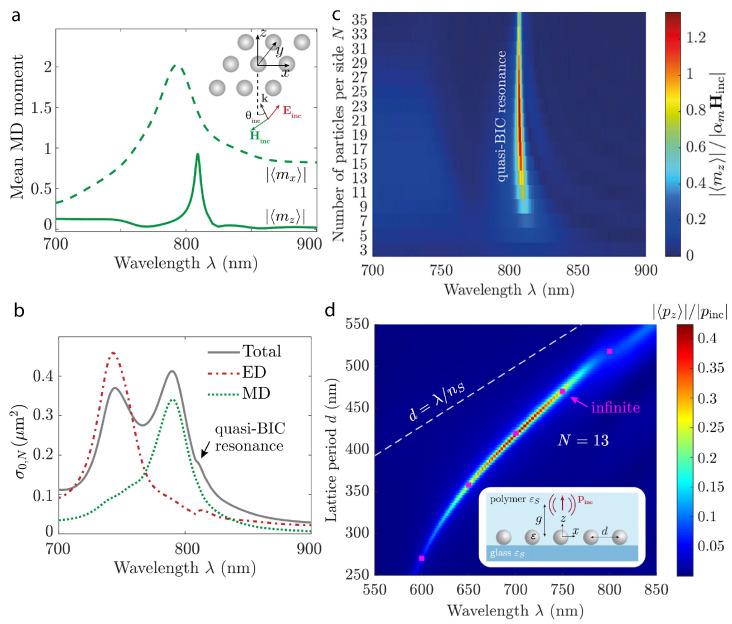
Effect of a finite-size nanoantenna array on quasi-BIC resonance and of average values of the metasurface multipolar components contributing to it. (**a**) Mean values of the MD moment for a nanoparticle array of finite size of 13×13. (**b**) Scattering cross-section per particle, as well as the ED and MD component contributions for a nanoparticle array of finite size of 13×13. (**c**) Normalized mean value of MD moment for a finite-size array of particles N×N. An obliquely incident plane wave impinges at the angle θ = 2°, and the lattice period of the nanoparticle array is *d* = 495 nm. (**d**) Normalized mean value of the ED moment as a function of *d* and operating wavelength λ. The highest value of the mean ED moment is related to the quasi-BIC excitation. The resonance is achieved by placing an ED 150 nm above the finite-size array while operating at the wavelength λ = 700 nm and *d* = 423 nm. (**a**–**d**) Reproduced with permission from [31]. Copyright 2024 by American Physical Society.

The detailed optical response of finite-sized metasurface arrays with silicon nanoparticles has been investigated with an emphasis on collective resonances [31]. The functionality of controlling light at the nanoscale, corresponding to the Kerker lattice effect and quasi-BICs under the influence of optical excitation, has been explored, including a reflection on an infinitely large number of spherical nanoparticles with a diameter of 200 nm in the periodic arrays. Nanoantennas are considered on the quartz glass substrate with a refractive index equal to 1.4 and covered with material of the same refractive index, and the spectral range of 620–900 nm is of interest. In this case, the contributions of an ED and an MD have been associated with the number of unit cells *N* and the lattice period of unit cell *d* (Figure 3). Modeling of the system response has indicated that the lattice Kerker effect becomes prominent for an array size between N≥9 and N≥30, saturating at the larger number of nanoantennas, enabling notable suppression of backward scattering. However, a quasi-BIC resonance appears from N≥11, and the quality factor of the resonance starts to degrade at N≥30.

The excitation of trapped mode was achieved using an analogous method of incorporating heterogeneity into the superstrate layer above a coherent metasurface made of periodic silicon disks [32]. The silicon nanodisks (array of 10×10) were fabricated on a quartz substrate with an inhomogeneous colloidal mixture superstrate covering them. This facilitates a strong electromagnetic coupling when the incident light influences the ED and MD excited in the silicon nanodisks. The inclusion of heterogeneity into the superstrate was controlled by the scaling factor of meta-atom design and the degree of heterogeneity. This affects the collective trapped-mode excitation and effective permittivity of the metasurface by introducing local asymmetry into the unit cells. The distinct presence of coplanar and non-coplanar components in the MD moment verifies the development of trapped modes inside the metasurface, as well as certain higher-order modes, which are perceived to be promising for spasers and nanolasers. A methodology for transforming accidental BICs into quasi-BICs has been established without affecting the symmetrical characteristics of the metasurface or the geometrical displacement of the composite unit cells [33]. The metastructures containing disk particles with refractive index = 2.45 and diameter = 200 nm, as well as spherical particles with refractive index = 3.5 and diameter = 250 nm, were examined in the visible range and normal incident light with a particular emphasis on multipolar moments of single parity. As the structural periodicity has been altered, the destructive interference of MD and ED moments results in BIC. However, the spectral position of pure quasi-BICs generated by the MQ resonance remains unaffected by the periodicity, but the quality factor becomes dependent on the metasurface period when it deviates from pure BIC criteria. Consequently, non-resonant BIC has been transformed into radiating quasi-BIC with a finite quality factor by incorporating a shift between the refractive index of the substrate and the configurable superstrate, which eventually makes them suitable for lasers, nonlinear applications, and sensing.

The properties of magnetic toroidal dipoles (MTDs), including distinct near-field patterns, resonance wavelength, confined electric fields, and strong Fano resonances, have been theoretically explored and experimentally realized [34]. Two silicon nanobars have been positioned in parallel to facilitate reverse vortex magnetic field distributions, successfully generating the head-to-tail ED and exciting the MTD mode in the space between the nanobars. The disruption of the metasurface translational symmetry has been governed by the offset distance, which is defined as the difference in length between the spacing of two silicon nanobars and their initial center-to-center spacing. It has been validated to play an important role in inducing the MTD and, as a result, setting up a guided mode resonance (GMR) inside the dielectric nanostructure, effectively trapping the electric field inside the layers. The GMR wavelength has been found to be almost uniform around 1474.5 nm, accompanied by a pronounced Fano resonance peak at the same wavelength due to precise offset distance modification. An analysis of both near-field distribution and far-field radiation has shown that the ED moments are oriented in the *XZ*-plane and the MTD has been activated along the *y*-axis, which accounts for 72.2% of the total scattered power radiated in the same direction. The fabrication metasurfaces have shown a high *Q*-factor of 5079, which has been attributed to the offset distance variations, and the subsequent MTD response can be used in developing nonlinear light sources and low-threshold lasers.

Recent research work has addressed the generation of third harmonics in all-dielectric metasurfaces sustaining dominant modes of ED, toroidal dipole (TD), and MD moments, as well as constructive interference of the aforementioned modes in the near-infrared spectrum [35]. Amorphous silicon nanopillars assembled in a square lattice with a periodicity of 850 nm and a height of 350 nm were embedded on a spin-on-glass substrate. The metasurface was irradiated with *x*-polarized light at a normal incidence, and the diameters of the nanopillars were modified to five distinct values of 260, 270, 320, 340, and 360 nm. Using this approach, the spectral distance was effectively regulated between the TD and MD modes. Numerical simulations have suggested a reflection dip at a wavelength of 1225 nm for diameters of 260 and 270 nm. At this wavelength, the constructive interference between the ED and TD moments contributed to the formation of a total electric dipole (TED), which acts as a super-dipole at the same wavelength. The constructive interference of the TED and MD modes was demonstrated to suppress backward scattering while simultaneously offering remarkable electric-field confinement stemming from the spectral overlap between the two modes. An impressive third-harmonic generation has been reported to occur when the TD and MD modes become spectrally identical at a diameter of 270 nm, and a maximum was achieved for 260 nm, corresponding to a 214-fold improvement compared to the 360 nm nanopillars with TD resonance. This makes the metasurface ideal for nonlinear up-conversion adaptations.

The forward machine learning (ML) model has been explored in recent work on predicting the scattering response of meta-atoms of any arbitrary shape [36]. This work further discusses an inverse-design model that generates geometrical specifications of meta-atoms with desired multipolar resonances and an ML model predicting the electric field distributions within the near-field area. The forward-predicting ML model has been specified based on a densely connected convolutional neural network. Specifically, the model includes 21 distinct wavelengths of data from arbitrarily shaped TiO_2_ meta-atom geometries for predicting the electric field distribution and induction of the first six multipolar moments. The predictions obtained with the preceding ML model were able to closely approximate the scattering cross-section and most of the features obtained with the finite element method simulation. As a metric, this has been verified by the root mean square error of $6.4\times 10^{-3}$ μm^2^ within the spectral range of 500–900 nm. Tandem inverse design, an ML model backed by a mean square error metric, has also been conceived to counter the concerns related to existence and uniqueness while harnessing the designed meta-atoms at the targeted wavelength with desired multipolar resonance specifications. A hybrid model combining the inverse design and the previously stated forward-predicting model has been adopted to more precisely estimate multipolar moments, which ultimately maps the meta-atom design utilizing feedback. Furthermore, a 3D electric field prediction model with a dense neural architecture has been implemented, along with a comprehensive analysis of the field interactions within the arbitrarily shaped meta-atoms. The geometry of the meta-atom undergoes six up–down conversions to generate a 3D electric field distribution. The developed inverse-design model has been employed to design two exemplary meta-atoms at two different wavelengths, implying that the optimal MD and MO resonance contribute up to 61% and 50% of the total scattering response. Furthermore, the outlined ML models can be helpful in modeling nanophotonic devices in both linear and nonlinear optical regimes.

The asymmetry known as chirality is traditionally a chemical domain, and the photonic field has embraced and harnessed it. The recent proposal has demonstrated an engineered 2D chiral structure composed entirely of non-handed plasmonic units (gold), a departure from traditional assemblies of chiral elements [37]. Circular dichroism, a measure of the differential absorption of left- and right-handed circularly polarized light, arises from the electromagnetic interplay between the constituent elements and the emergence of collective lattice resonances. Furthermore, a nanoantenna array composed of highly conductive layered MXene material has been designed, leveraging the lattice’s distinctive properties to tailor the optical response [38]. The chiral characteristics of this periodic MXene nanoantenna array have been demonstrated, with these properties governed by the lattice periodicity. Rectangular lattices, exhibiting anisotropy, demonstrate substantially amplified circular-dichroism signals, while square lattices display attenuated chiral signatures. By judiciously adjusting geometric features and refractive indices, the precise tailoring of the circular-dichroism spectral peak and bandwidth is achievable, paving the way for advanced polarization filtering devices and sensitive chiral biosensing technologies.

An alternative strategy to influence the ability to interact differentially with left and right circularly polarized light has been evaluated by tuning the dipole and multipolar resonance of a silicon dielectric nanostructure [39]. Significant coupling of the ED and EQ terms has resulted in near- and far-field chiroptical properties, such as the super-chirality of the silicon metasurface, which has been achieved by simply altering the nanostructure height. S-structured unit cells have been assembled periodically into a square structure of enantiomorphs and racemic arrays with a lattice period of 850 nm and fabricated with four distinct heights of 160, 180, 210, and 240 nm. For *x*-polarization, the increase in reflectance for the 240 nm design has also been replicated in the total scattering cross-section, with a considerable influence being induced by the MQ component. Within the desired spectrum, the total scattering cross-section for *y*-polarization has demonstrated significant contributions from both the MD and EQ components. The greater MQ contributions for the thicker sample have been explained by the fact that the magnetic components of the resonances are parallel to the incident light, as well as the thicker sample’s ability to maintain multipolar moments. Multipolar expansion has been employed to demonstrate the effective contribution of the ED and EQ terms to optical activity and chiral asymmetry in the near fields. The dielectric metasurface has been proposed as a novel design for detecting enantiomeric or bio-macromolecular compounds.

By manipulating the array period, collective lattice resonances can be readily tuned across a wide spectral band. This tunability is remarkably independent of the individual nanoparticle’s material, dimensions, or morphology, enabling highly adaptable optical designs. Consequently, the resonant behavior can be optimized without the constraints imposed by the inherent properties of the constituent nanostructures. Strong resonances have been demonstrated in arrays of such nanoparticles with significant optical losses, highlighting their potential for effective light manipulation in ultra-thin optical components [21]. The nanostructure design that enhances the generation of plasmonic hot electrons from periodically arranged gold nanoelectrodes has been successfully demonstrated [40]. This design optimizes the interaction between incident light and localized surface plasmons, leading to efficient hot carrier generation.

Sharp collective excitations in nanoparticle lattices through EQ and MD resonant interactions can result in significantly higher linear resonance. In addition, second-harmonic generation (SHG) near the array resonance associated with various nanoparticle multipoles has also been shown to be substantially enhanced and effectively tuned by adjusting the lattice [41]. Mie theory can be used to calculate the absorption, scattering, and extinction cross-sections for an isolated spherical nanoparticle [42], but the analysis of more complex shapes requires the use of numerical simulations and subsequent decomposition of the induced optical field into multipolar components [11,43,44]. Multipolar decomposition has been extensively employed to examine the contribution of multipoles in resonance excitation, radiation, and the scattering of electromagnetic waves. The precise multipolar decomposition for a spherical nanoantenna in the periodic lattice has been shown to closely match numerical simulations near lattice resonances [45]. It has been demonstrated that only a limited set of multipoles is necessary to accurately describe the outcomes.

Recent research has introduced a metasurface composed of optical nanoantennas fabricated from the van der Waals material hexagonal boron nitride [46]. In this metasurface, the excitation of multipolar resonant modes leads to directional scattering and a complete suppression of reflectance, demonstrating the resonant Kerker effect. Van der Waals nanoantennas are expected to support localized resonances, making them promising functional elements for metasurfaces and transdimensional photonic devices. High-refractive-index nanoantennas confine light at subwavelength scales via Mie resonances and have been widely explored for photonic applications. Building on this, van der Waals transition metal dichalcogenides offer a new paradigm with tunable optical properties, strong anisotropy, and equally high refractive indices. Unlike conventional high-refractive-index materials, transition metal dichalcogenides enable the dynamic control of metasurfaces and ultrathin optics, exhibiting strong spectral responses in the visible and near-infrared regions, with properties governed by nanoantenna size and arrangement. A periodic arrangement of disk-like nanoantennas composed of a TMDC material, tungsten disulfide WS_2_, positioned atop a silicon layer and oxide substrate, has been explored [47]. The resonance of the TMDC disk-shaped nanoantenna array has been demonstrated to be influenced by changes in the silicon layer thickness and is dependent on the presence of an index-matching superstrate cover.

To wrap up this part, multipolar resonances in metasurfaces have great potential to optimize light–matter interactions at the nanoscale by providing precise control over the scattering, absorption, and transmission properties of nanoantennas and other building blocks. Highly efficient dynamic and reconfigurable metastructures can be engineered for optical applications utilizing electric, magnetic, or higher-order multipolar modes, as well as multilayer designs with reconfigurable properties. Integrating these multipolar metastructures with quantum photonics can also contribute to significant advances in novel technologies, as well as the development of sensing and photovoltaics. Capitalizing on advances in highly efficient nanoantennas and ultra-thin metalenses, one can expand the nanophotonic capability to transform beam steering, communication, and quantum technology.

## 3. Spontaneous Emission and Light–Matter Interaction

The Purcell factor (PF) quantifies the degree to which the spontaneous emission rate of a dipolar emitter is enhanced when it is placed in a resonant environment relative to its emission rate in free space. The enhancement arises from modifications to the local density of optical states (LDOS), which determines how efficiently an emitter can couple to the surrounding optical modes. Multipolar resonances in nanostructures shape LDOS, thereby influencing spontaneous emission rates through Purcell enhancement or suppression. This control over light–matter interaction enables tailored emission properties, which are crucial for applications in quantum optics and nanophotonics.

In hybrid-lattice devices composed of colloidal QDs and plasmonic nanoantennas, the so-called colloidal–QD–plasmonic systems, the coupling intensity has recently been shown to vary from weak to strong by adjusting the system parameters [48]. By tuning colloidal QD concentrations, Purcell-enhanced emission at low densities and the emergence of polariton states at higher densities have been observed.

All-dielectric nanostructures generally exhibit lower PFs compared to plasmonic nanostructures because of the considerable energy loss through radiation and the selective confinement of light energy within the dielectric material instead of the surfaces. However, recent theoretical and experimental advances have led to substantial improvements in PFs for all-dielectric nanostructures [49]. In particular, a finite-size chain of silicon nanoparticles yields a strong Purcell effect, with theoretical predictions of two orders of magnitude of enhancement in PF. Eigenmode analysis connects this enhancement to a van Hove singularity in the density of states. A concept-validation experiment at microwave frequencies confirmed these predictions, showing a 65-fold increase in the PF in a 10-dielectric-particle-long chain. In general, all-dielectric nanostructures support multipolar resonances that modify the local electromagnetic environment, enhancing or inhibiting spontaneous emission via the Purcell effect. This resonant control over light–matter interaction enables the efficient manipulation of emission dynamics for applications in quantum photonics and nonlinear optics.

A comparative analysis has been performed for various dielectric nanostructures to optimize the excitation and emission properties of a single CdSe/ZnS QD incorporated in the design [50]. Different geometries have been evaluated based on electric field enhancement, radiation efficiency, and the PF across the visible spectrum, including homogeneous nanoparticle dimers (composed of either metals, such as gold, or dielectrics, such as silicon or moderate-refractive-index (MRI) materials) and hybrid dimers (combining gold and MRI, MRI and silicon, or gold and silicon nanoparticles). It has been observed that the photoluminescence enhancement of CdSe/ZnS QDs coupled with MRI dielectric particles can be attributed to the efficient interaction with light over a wide range of wavelengths. However, hybrid dielectric nanostructures that integrate silicon and gold provide a stronger emission intensity and higher PFs, benefiting from the combined effects of strong plasmonic field enhancement and Mie resonances. These insights can be further extended to inform the design of advanced single-photon sources.

Coupling a localized emitter with a hybrid system composed of a plasmonic dipolar nanoantenna and a silicon nanorod has been reported to achieve unidirectional emission [51]. The plasmonic antenna enhances the emitter’s radiative decay rate, while the silicon nanorod redirects the emitted light into a single direction by exciting multipolar modes within the nanorod. This hybrid antenna exhibits high forward directivity, with a front-to-back ratio of 30 dB, and operates across a broad spectral range in the visible domain, covering a bandwidth of 240 nm.

Plasmon-enhanced spontaneous emission and strong light–matter coupling are of great interest in hybrid systems that integrate nanoparticles and quantum optical emitters [52]. Plasmonic nanostructures function as optical nanoantennas, enhancing and concentrating optical fields while increasing the LDOS. This enhancement increases the fluorescence intensity and reduces the lifetime of the emitter. Plasmonic nanogaps create extreme field confinement and small mode volumes, leading to significant modifications in spontaneous emission. By precisely engineering these nanostructures, the directionality and polarization of the emission spectrum can be tailored, improving the fluorescence collection efficiency and enabling long-range energy transfer between quantum emitters. However, plasmonic nanowaveguides enable the propagation of signals at scales smaller than the diffraction limit of light, making them essential components in quantum plasmonic circuits. In particular, when plasmons and excitons couple coherently, they can give rise to novel hybrid states, such as plexcitons, which have been observed in QDs, J-aggregates, and 2D transition metal dichalcogenides interacting with various plasmonic nanostructures. Strong plasmon–exciton coupling interactions lead to novel optical excitations, such as ultra-fast Rabi oscillations. The coupling strength is highly sensitive to factors such as emitter placement, dipole orientation, and spectral matching. Various fabrication techniques, such as molecular linker-assisted self-assembly, focused ion beam milling, electrohydrodynamic nano-printing, and electron beam lithography, have been developed to enable efficient engineering of hybrid systems, supporting controlled emission and tuned coupling strength.

Advancing plasmon–exciton interactions requires the development of materials with greater resistance to photodamage, refined nanostructure designs, and improved assembly methods. Enhancing key emission characteristics, such as brightness, decay rates, and quantum yield, can unlock broader applications in optoelectronics and quantum technologies. While a strong coupling level has been successfully achieved, further investigation is required to advance into the realm of ultra-strong coupling and to explore cavity quantum electrodynamics at the nanoscale. Continued advances in nanotechnology and materials science can pave the way for new breakthroughs in plasmon–exciton interactions and their integration into quantum photonic and nanophotonic applications.

A quasinormal-mode theory has been proposed that accurately describes light–matter interactions in coupled loss–gain resonators across various spatial points and frequencies [53]. Moreover, a quasinormal-mode-based coupled-mode theory has been developed, which intuitively models bare resonators within a non-Hermitian framework, providing accurate eigenvalues and modes while enabling efficient control over light–matter interactions in loss–gain systems. The study analytically demonstrated that the line shapes of spontaneous emission exhibit spectral behaviors resembling Lorentzian and Lorentzian-squared profiles. Moreover, when the resonators with coupled modes are closely spaced, the numerical solutions for microdisks show a much more complex spectral behavior, including negative PFs. Overall, this motivated the development of a more advanced model than the classical dipole model to explore the behavior of coupled loss–gain resonators.

To achieve sophisticated optical functions, the precise fabrication of thin films and multilayer structures is essential. Nanostructured films, integral to meta-optics and surface engineering, allow for the nanoscale manipulation of optical properties. The accurate control of film thickness and material characteristics results in strong mode confinement and tunable support for specific optical modes, which are critical for optimizing resonance and device performance [54,55,56].

Surface plasmon polaritons (SPPs) originate from the coupling of incident optical waves with the collective electron excitations, which propagate along the interface of a metal (or doped semiconductor) and a dielectric. This interaction gives rise to coherent optical waves that are tightly confined to the interface and propagate along it while decaying evanescently into both media. A comparative analysis has been conducted on several metallic materials, examining how their LDOS and PF differ with frequency. As shown by the numerical results, silver exhibits a higher PF at its resonant frequency, facilitating enhanced light-collection efficiency from the emitter and accelerating the decay rate. Further analysis of the variation in the PF of silver with distance from the surface, as well as in the presence and absence of the dielectric coating, has been carried out [57]. The LDOS has been revealed to decrease as the distance from the metal surface increases and eventually approaches the value in a vacuum because of the confinement of the plasmonic field. Additionally, the dielectric coating decreases the resonant frequency of the PF, and introducing a high dielectric constant coating can effectively enhance the PF.

Metal–dielectric antennas designed to overcome the limitations of purely metallic optical antennas have been reported [58]. Specifically, the design addresses the trade-off between spontaneous emission enhancement and low radiative efficiency, which arises due to Ohmic losses and electron surface collisions in metallic antennas. By incorporating nanoscopic dielectric structures at the tips of the antenna, the proposed design achieves both high enhancement and high radiative efficiency simultaneously. The antenna enhances the spontaneous emission rate by a factor of $5\times 10^{5}$ compared to the one in the free space. Systems with a radiative efficiency of 70% significantly outperform all-metal antennas with comparable enhancement. Furthermore, the study addresses the challenge of defining a clear mode volume for metallic antennas, which makes the calculation of the PF based on the LDOS not directly applicable. To resolve this, a new formula for the effective mode volume $V_{\mathrm{eff}}$ has been proposed

$$V_{\mathrm{eff}}={\left(\frac{3}{4\pi^{2}}\right)}^{2}d^{2}\lambda {\left(\frac{\lambda }{l}\right)}^{5},$$

where *l* is the characteristic length scale related to the antenna geometry, λ is the light wavelength, and *d* is the size of the antenna. This formula enables the PF analysis of metallic antennas, providing a more unified framework for understanding and designing nanophotonic devices that combine metallic antennas with quantum emitters.

**Figure 4:**
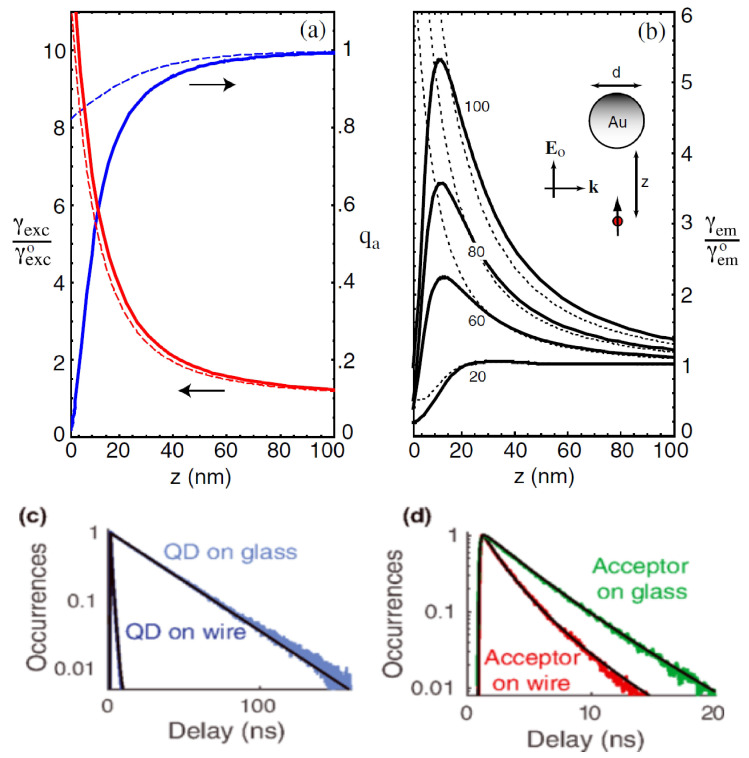
(**a**) Quantum yield $q_{a}$ (shown by arrow to the right) and normalized excitation rate $\gamma_{\mathrm{exc}}$ (shown by arrow to the left) and (**b**) normalized fluorescence rate $\gamma_{\mathrm{em}}$, all plotted against the distance *z* separating the molecule and the particle. Both excitation and fluorescence rates are normalized to their free-space values (i.e., at z→∞). Solid curves are generated using MMP calculations (maximum error 2%), whereas dashed curves correspond to dipolar approximation, which is inaccurate at close molecule–particle proximity. The particle diameter in (**a**) is *d* = 80 nm, and the ones in (**b**) are as labeled. The excitation wavelength is λ = 650 nm, and the permittivity of gold is ε=−12.99+i1.09. Reproduced with permission from [59]. Copyright 2006, American Physical Society. (**c**) Comparison of fluorescence decay for a single QD on glass (light blue) and on the wire (dark blue). (**d**) Comparison of fluorescence decay for the acceptor on glass and on the wire under plasmon excitation. (**a**–**d**) Reproduced with permission from [60]. Copyright 2017, American Physical Society.

A gradual shift from fluorescence enhancement to quenching is experimentally observed in a system consisting of a single molecule that interacts with a laser-excited gold nanoparticle, as further validated by theoretical methods [59]. Multiple multipole (MMP) and dipolar approximation methods have been used to evaluate fluorescence rates as a function of the separation *z* between the nanoparticle and the molecule (Figure 4a,b). As shown in panel a, the validity of the dipolar approximation in the description of the excitation rate works only when the particle size *d* is much smaller than the wavelength of light under consideration. In this case, the field used for excitation is assumed to be homogeneous throughout the particle (interactions occur in the far-field regime). However, for the quantum yield, as shown in panel b, the MMP method demonstrates that at short distances, the decrease in quantum yield surpasses the rise in excitation rate, leading to fluorescence quenching. In contrast, the quantum yield is strongly overestimated by the dipolar approximation, which is insufficient for describing fluorescence quenching at short distances. Furthermore, fluorescence enhancement is optimized at red-shifted wavelengths relative to plasmon resonance, with 680 nm providing the strongest enhancement. Both local field enhancement and nonradiative energy transfer play critical roles in determining the fluorescence behavior.

Time-correlated emission has been investigated for the case when the system is composed of a single CdSe/CdS/ZnS QD and a fluorescent nanobead separated by several micrometers [60]. A silver nanowire acts as a bridge for energy transfer between the QD and the nanobead. The QDs generate individual plasmons on the nanowire, and these plasmonic excitations propagate and transfer energy to excite the fluorescent nanobead. The linear correlation between the photon emissions of the QD and the nanobead reveals that the molecules within the bead exhibit blinking behavior identical to that of the QD. Further investigation has been conducted to explore the influence of the local environment (e.g., the nanowire) on the emission properties of the QD. As shown in Figure 4c, the fluorescence decay rate Γ, at which an excited QD relaxes to its ground state through photon emission, has been determined by examining the decay histogram. The histogram records the time delay between excitation and photon emission events. The fluorescence decay rate of QD on the glass substrate follows a single exponential decay function with a measured decay rate of 0.034 ns^−1^, which corresponds to a relatively long excited-state lifetime. In contrast, the decay of the QD on the nanowire follows a double exponential decay function, indicating the presence of two distinct decay processes. Approximately 80% of the emission exhibits a decay rate of 0.67 ns^−1^, which is significantly lower than that observed on the glass substrate. A higher rate of over 12 ns^−1^ is also observed and is believed to be caused by the significant formation of biexcitons in the QD, with a biexciton-to-exciton ratio of around 30%. The fluorescence decay of the acceptor bead, which is excited by a QD-launched plasmon, has also been characterized (Figure 4d). By analyzing the decay rates of the acceptor on both a glass substrate and a silver nanowire, it is evident that plasmonic coupling significantly enhances the decay rate of the acceptor, with a PF of ∼2. Moreover, the decay rate increases further when the acceptor is excited through the surface plasmon due to the excitation of all molecules within the bead. This demonstrates the importance of plasmonic interactions in enhancing fluorescence in nanoscale systems.

**Figure 5:**
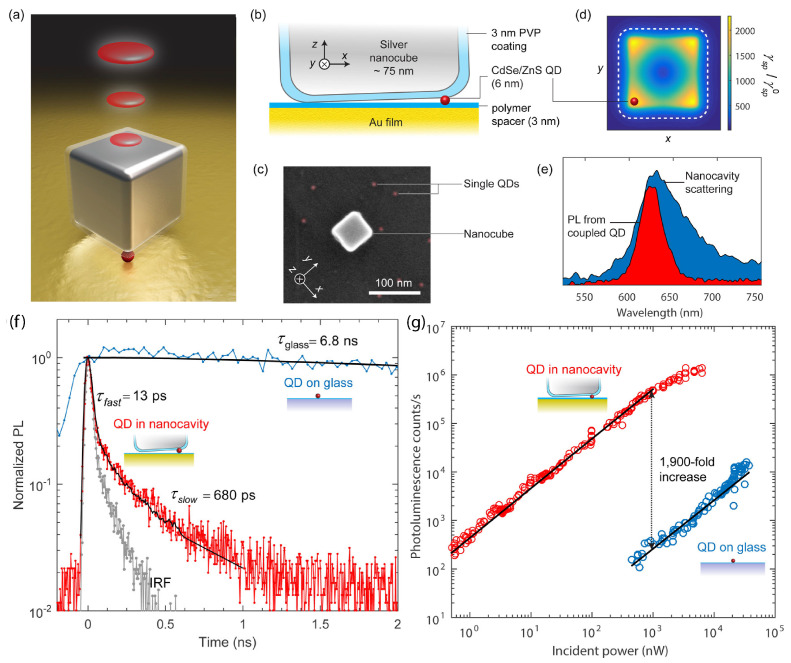
(**a**) Schematic of single-photon emission from an individual colloidal QD in the gap between an Ag cube and a gold film. (**b**) Cross-sectional schematic of an individual QD embedded in the cavity. The QD with a ∼6 nm diameter is positioned near one of the corners of the cube, at which the field enhancement is maximal. (**c**) Scanning electron microscope image of the sample, showing individual QDs and a single cube. Some cavities contain an individual QD in the gap between the cube and the gold film, which is invisible through scanning electron microscopy. (**d**) Simulated enhancement in the spontaneous emission rate, normalized to the free-space emission rate of a randomly oriented dipole, plotted versus the lateral position within the gap. An individual QD is schematically depicted near the corner, and a rate enhancement of ∼2000× is expected. (**e**) Measured scattering spectrum of a single cavity (light blue), with a primary resonance shown at $\lambda_{\mathrm{np}}=630$ nm. Photoluminescence (denoted ‘PL’) from a single QD coupled to the same antenna (red) exhibits an emission spectrum that overlaps well with the plasmonic resonance. (**f**) Time-resolved photoluminescence (denoted ‘PL’) measured for a single QD adjacent to a cavity (red). It displays a bi-exponential decay, which is characterized by a rapid decay time of $\tau_{\mathrm{fast}}=13$ ps and a slower decay time of $\tau_{\mathrm{slow}}=680$ ps. The gray curve represents the limitation imposed by the instrument response function (the curve is denoted ‘IRF’ on the plot) of the avalanche photodiode detector on the fast component. (**g**) The relationship between photoluminescence counts and excitation power shown for a single QD combined with a cavity (red) and compared to counts for a single QD on glass (light blue). The photoluminescence intensity exhibits a linear dependence on incident excitation power for excitation powers lower than 1000 nW in the nanocavity-coupled system. In the linear region, the coupled QD achieves a maximum count rate of 1 MHz. In comparison to the single QD on glass under comparable excitation conditions, the nanocavity-coupled QD demonstrates an emission intensity enhancement of 1900 times. (**a**–**g**) Reproduced with permission from [61]. Copyright 2016, American Chemical Society.

Ultra-fast spontaneous emission is achieved from a quantum emitter in strong coupling with a nanocavity at ambient temperature [61]. The nanocavity consists of a silver nanocube and a gold film, which are separated by a 12 nm thick gap, with 3 nm thick polymer layers sandwiching the single colloidal QD inside the gap (Figure 5, panels a–c). Full-wave simulations demonstrate that the Purcell enhancement varies spatially across the nanocavity, with the enhancement magnitude decreasing from the corners to the center of the nanocube, as shown in Figure 5d. These enhancements occur at the primary resonant mode of the nanocavity, characterized using white-light scattering spectra. Furthermore, the measured scattering spectrum of the single antenna shown in panel e reveals a resonance wavelength of 630 nm. Thus, QDs with an emission spectrum centered at 630 nm are selected, ensuring optimal spectral overlap. The cavity serves as an effective light-harvesting nanoantenna, significantly enhances the total emission intensity, and reduces the emission lifetime while also enabling directional light emission concentrated into a single lobe perpendicular to the surface. The fabrication process is statistical, but future advancements could enable the precise positioning of QDs for optimal coupling.

Figure 5f shows the lifetime of the single-photon emitter determined through time-resolved emission dynamics measurements. For the nanocavity-coupled QD case, the measurements exhibited a bi-exponential decay profile, characterized by a rapid decay component with a lifetime of $\tau_{\mathrm{NPA}}=13$ ps (constrained by the instrument response function of the detector), as determined by fitting the data. The analysis showed that 97% of the photons are released during the fast decay phase, indicating that most of the emission occurs on an ultra-fast timescale. The slower decay component is likely due to emission dipoles in the QD that are not optimally aligned with the primary electric field within the nanocavity, resulting in less efficient coupling. Contrary, a single QD on glass exhibits a photoluminescence decay lifetime of $\tau_{\mathrm{glass}}=6.8$ ns. The measured decay lifetime of 13 ps sets a detector-limited lower bound for the PF, which is 540 for a single QD adjacent to the cavity. The radiative rate enhancement for the coupled QD is related to the intrinsic quantum yield of QDs on glass, which is ≈20%, and the quantum yield of the cavity, which is ≈50%.

The time-averaged emission rate measurement results, as shown in Figure 5g, reveal a linear correlation between excitation power and emission rate for the coupled QD system at incident powers lower than 1 μW. Specifically, at an excitation power of 1 μW, the detected count rate on the single-photon detector reaches 500 kHz. This linear dependence indicates that the system operates in a regime where the emission rate is directly proportional to the excitation power, a characteristic behavior of single-photon emitters under low excitation conditions. In contrast, the photon rate of a typical QD on glass under the same excitation power is significantly lower, which is only 260 Hz.

Multiperiodic metal–dielectric multilayers constitute hyperbolic metamaterials that are structured with unit cells consisting of two distinct dielectric layers alternately stacked with uniform metallic layers [62]. A systematic investigation of the properties of multiperiodic hyperbolic metamaterials and the frequency dependence of the PF for different emitter placements has been reported. Focusing on a novel biperiodic structure with a four-layer unit cell that comprises two distinct dielectric layers alternating with two identical metallic layers, the study reveals that dipolar radiation primarily couples to SPPs at nearby metal–dielectric interfaces. Additionally, the enhancement of the PF is attributed to the coupling of emitted light to both SPPs and volume plasmonic excitations within the multilayer metamaterial. The spectral redistribution of the PF, combined with the enhanced spontaneous emission that exceeds that of conventional periodic structures, highlights the promising potential of multi-periodicity as an alternative strategy that complements the known effect of the thinning dielectric layer in metal–insulator–metal structures.

Dielectric materials exhibit much lower absorption losses compared to metallic materials, making them a more efficient alternative to enhance light–matter interactions [63]. A significant increase in the strength of the electromagnetic field has been reported to occur in the vicinity of identical pairs of dielectric nanoparticles (i.e., dielectric homodimers). This enhancement arises from Fano-like resonances, which result from the interference between electric and magnetic multipolar modes. Combined with the low-loss environment provided by dielectric materials, such near-field enhancement can substantially increase the LDOS around quantum emitters. This is predicted to lead to a considerable improvement in PF, potentially exceeding values of 10^3^. Furthermore, Fano-like resonances are highly sensitive to the dimensions of the particles and the separation distance between them. As a result, the resonance wavelengths can be easily tuned by adjusting the size of the nanoparticles or reducing the gap distance between the two dielectric nanoparticles. This tunability makes dielectric homodimers highly versatile for applications in nanophotonics, such as single-photon sources, sensors, and enhanced light–matter interaction platforms.

To achieve multi-resonant metasurfaces and enhanced PF in the visible range, an approach has been established that utilizes lattice-induced coupling effects between high-order multipoles in low-optical-loss dielectric nanostructures in such a spectral range. The metasurface consists of 2D arrays of uniformly arranged nanodisks with fixed particle and lattice configurations [29], and its resonance behavior is governed by energy exchange among groups of Mie-type multipoles of the same parity. Additionally, triple resonance near the red, green, and blue wavelengths can be achieved by modulating the period of the metasurface with a square lattice accordingly potentially enhancing the emission.

A spin–photon interface that facilitates the coherent and efficient transfer of quantum information between the spin of a particle and a photon is realized with an erbium-doped nanophotonic silicon resonator [64]. This erbium-doped resonator provides a platform for storing quantum information in the spin of erbium ions and efficiently transferring it to photons, enabling spin-selective optical transitions. The spin-resolved excitation of individual erbium dopants in silicon achieves a narrow spectral diffusion linewidth of less than 0.1 GHz, indicating great coherence and stability for quantum operations. The optical Rabi oscillations and a well-coupled single emitter with a 78-fold Purcell enhancement compared to that in bulk silicon. A single-photon nanoantenna consists of a single quantum emitter coupled to a nanoantenna in its near field [65]. The nanoantenna enhances light–matter interactions by coupling photons into localized surface plasmon resonances, creating a high LDOS in subwavelength volumes. This LDOS enhancement, which exceeds the diffraction limit, increases the emitter’s spontaneous emission rate via the Purcell effect and enables precise control over photon emission properties, such as directionality and efficiency. Recently, significant advancements in this field have enabled an enhancement of brightness and PFs that was several orders of magnitude.

An axisymmetric silicon nanoresonator featuring a tapered angle well has recently been reported [66]. This design enables efficient coupling with MD emitters placed in the well, significantly enhancing the decay rate of the emitters and leading to a PF of approximately 500, which substantially surpasses that of a solid silicon nanosphere of comparable size. This enhancement is attributed to the resonant coupling between the MD emitter and the resonator’s MD mode. Furthermore, when the resonator is supported by a metallic substrate, spontaneous MD emission is further enhanced, with the PF exceeding $10^{3}$. This additional enhancement results from a quadrupolar high-Q mode generated by image–charge interactions in the nanoantenna. The geometric dependencies of the PF and the resonance frequency have been thoroughly analyzed. It is observed that the PF decreases with an increase in the dimensions of the perforated silicon sphere in air, the well volume, and the distance of the MD emitter from the center of the sphere. The ability to tailor the resonator’s properties through geometric adjustments, combined with the significant enhancement achieved, makes this design highly promising for future chip-scale nanophotonic applications.

When considering metasurface materials to enhance the Purcell factor, plasmonic and dielectric metasurfaces exhibit distinct advantages and limitations. Plasmonic metasurfaces, typically composed of noble metals such as gold and silver, provide extreme field confinement and strong LDOS, leading to substantial spontaneous emission enhancement. However, their high intrinsic losses due to Ohmic dissipation limit their efficiency and applicability in low-power or quantum optics applications. In contrast, dielectric metasurfaces, made from materials such as silicon or titanium dioxide, support high-quality factor Mie resonances with significantly lower energy dissipation, enabling strong Purcell enhancement without excessive losses. Additionally, all-dielectric metasurfaces allow for efficient integration with photonic circuits and can support BICs, further improving emission control. Although plasmonic structures excel in subwavelength-mode confinement and nonlinear interactions, dielectric metasurfaces offer superior efficiency and scalability, making them more suitable for applications requiring low-loss and high-*Q* resonances, such as quantum emitters and optical sensing.

It is important to note that many structures capable of achieving strong field enhancement do not necessarily result in efficient light emission. This discrepancy arises because the mechanisms driving field enhancement, such as localized surface plasmon resonance and extreme field confinement, often involve significant non-radiative losses, which can quench emissions rather than enhance them. A silicon nanoantenna array has been designed with unit cells consisting of four basic square elements: one larger square antenna element accompanied by three smaller square elements. Photoluminescence enhancement has been achieved in this structure through three key aspects (trifecta enhancements): resonant absorption of pump laser energy, a Purcell-effect-driven increase in radiative decay rates, and directional radiation from molecules embedded in the hybrid nanoantenna array [67]. The array features nanogaps of approximately 10 nm, enabling a remarkable 1200-fold photoluminescence enhancement and a PF of ∼47. The design leverages Mie resonances to achieve these enhancements, with simultaneous improvements in absorption, radiative decay rates, and directionality. Specifically, the Rhodamine 6G molecules exhibited a higher quantum yield of ∼50%, compared to their plasmonic counterparts.

To summarize this discussion of enhanced emission, one can note that the ongoing progress in the Purcell factor highlights the potential of hybrid nanostructures, all-dielectric systems, and multi-resonant metasurfaces to significantly enhance light–matter interactions. These advances hold promise for applications in quantum optics, single-photon sources, and nanophotonics. However, challenges such as non-radiative losses and fabrication precision need to be overcome to fully harness these developments.

## 4. Metasurface-Enhanced Photodetection and Sensing

Metasurfaces have emerged as a transformative technology for enhancing photodetector performance by enabling the precise manipulation of light at the nanoscale. In particular, multipolar resonances in complex nanoantennas can enhance photodetection in metasurfaces by tailoring light–matter interactions for improved absorption and charge carrier generation. Recent research has demonstrated their ability to significantly improve sensitivity, broaden detection ranges, and facilitate multifunctionality by controlling various light–matter interactions [68]. These materials offer advancements in applications such as holography, biosensing, and imaging, contributing to miniaturization and improved detection capabilities. By optimizing light absorption through mechanisms such as plasmonic and Mie resonances, metasurfaces have proven particularly valuable in cutting-edge fields, including quantum computing, high-speed communication, and autonomous systems [69]. By leveraging multipolar resonances in complex nanoantennas, metasurfaces can achieve superior photodetection through enhanced light harvesting and tailored electromagnetic responses.

Integrating metasurfaces with photodetectors can enhance pixel-level light interaction, allowing the advanced detection of light and its characteristics, such as the polarization, phase, and wavelength [70]. In addition to this, metastructures, which are engineered nanostructures with properties not found in nature, offer additional avenues for innovative photodetection approaches. Examples include hybrid structures such as graphene–metastructure hybrids, patterned nanostructures, and van der Waals materials, which enhance functionality and performance [71]. They enable more versatile detection capabilities by expanding operational bandwidths and improving sensitivity. Furthermore, metasurfaces and, more generally, metastructures hold potential for next-generation technologies by addressing challenges related to miniaturization, multifunctionality, and detection efficiency. Their capability to control light at the subwavelength level makes them essential for driving future innovations in photodetector technology.

A novel giant plasmonic photogalvanic effect has been predicted in semiconductor media containing aligned, noncentrosymmetric metallic nanoclusters [72]. The asymmetric nanoparticle geometry enables directional photoelectron emission and a resultant photocurrent under uniform illumination, vastly exceeding the conventional bulk photovoltaic effect. In standard photodetection (e.g., in photodiodes or photoconductors), photocurrent is typically generated by carrier excitation across a bandgap followed by drift or diffusion under an external bias. In contrast, the plasmonic photogalvanic effect arises from asymmetric photoelectron emission due to the nanoparticle’s noncentrosymmetric shape, creating a spontaneous photocurrent without an external bias. Although photodetectors convert light into electrical signals for imaging, sensing, and communication, this effect could lead to novel bias-free photodetection mechanisms or even ultra-sensitive light-driven electronics leveraging plasmonic nanostructures.

Metasurface-enhanced photodetectors have emerged as a transformative technology that offers unprecedented control over light–matter interactions for improved detection efficiency, spectral selectivity, and compact integration. By leveraging nanoscale resonances, innovative designs have been demonstrated to surpass the performance of traditional photodetectors across various applications. These advances have broadened the horizons for optoelectronic devices, allowing the realization of more compact, efficient, and versatile photodetection systems.

**Figure 6:**
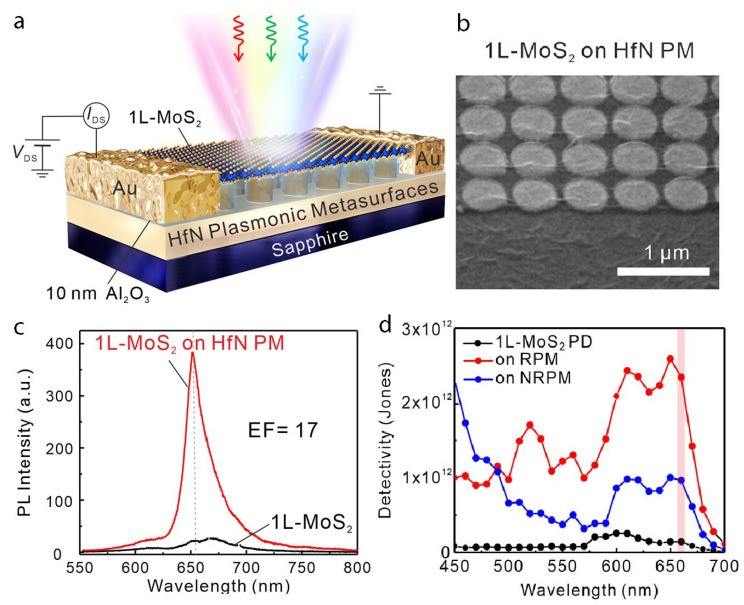
(**a**) Photodetector schematic that includes the integrated HfN plasmonic metasurface covered with a 10 nm Al_2_O_3_ dielectric film and a monolayer crystalline MoS_2_. (**b**) Forty-five-degree-tilted-angle SEM image of the HfN plasmonic metasurface after being covered by a single layer of MoS_2_. (**c**) Photoluminescence PL intensity enhanced by the HfN metasurface and monolayer MoS_2_ (red), exhibiting a 17-fold improvement at a wavelength of 670 nm. Monolayer MoS_2_ effect without the metasurface is shown for comparison (black). (**d**) Spectral detectivity as a function of wavelength for monolayer MoS_2_ photodetectors integrated with resonant plasmonic nanostructures (red), non-resonant plasmonic nanostructures (blue), and pristine monolayer MoS_2_ photodetectors (black). (**a**–**d**) Reproduced with permission from [73]. Copyright 2024 by American Chemical Society.

Significant advances in detection and sensing technologies have been achieved through the integration of monolayer molybdenum disulfide with hafnium nitride plasmonic metasurfaces, dramatically enhancing light absorption and photocurrent generation (Figure 6). The resonant electromagnetic field created by the metasurface substantially amplifies the photogating effect, resulting in an extraordinary 118-fold increase in photocurrent [73]. Additionally, the MoS_2_/Al_2_O_3_/HfN heterostructure contributes an additional 17-fold photogating gain, further boosting device performance. Including an insulating Al_2_O_3_ layer effectively suppresses dark current, achieving remarkably low 8 pA values and an impressive detectivity of $2.58\times 10^{12}$ Jones. This innovative integration supports scalable wafer-level fabrication processes, enabling advanced optoelectronic applications in imaging, sensing, and optical communication systems.

Hybrid gap–plasmon metasurfaces have also been demonstrated to improve MoS_2_ photodetectors, addressing limitations such as weak light–matter interaction and surface trap states [74]. These metasurfaces create strong field confinement, enhancing light absorption and reducing trap-state effects. The device exhibits a 30-fold polarization sensitivity and sufficient performance for perpendicular polarization. The linear photocurrent–voltage relationship and the high-frequency response highlight its suitability for rapid optical signal detection. This approach offers a promising direction for advanced 2D material-based photodetectors.

Dynamic color filtering and adaptive photodetection have been confirmed through antimony trisulfide nano-gratings. These metasurfaces feature a subwavelength antimony trisulfide grating deposited on a silver layer, achieving near-unity absorption and significantly enhancing photocarrier collection [75]. The tunability of these devices arises from thermally induced phase transitions and polarization-dependent optical responses, allowing for the excitation of both Fabry–Perot and surface plasmon resonances. This dynamic adaptability enables the metasurface to transition into an adaptive photodetector, leveraging strong light absorption and efficient carrier collection to generate distinct polarization-sensitive photocurrent responses. These multifunctional metasurfaces show great potential for future applications in tunable optical systems, including advanced imaging, optical communication, and sensing technologies.

Furthermore, Sb_2_Te_3_ metasurfaces have been investigated for ultra-broadband photodetection, leveraging both optical and thermoelectric properties. Sb_2_Te_3_ supports interband plasmonic resonances in the visible range and Mie resonances in the mid-infrared (mid-IR), while its high Seebeck coefficient enables the efficient conversion of absorbed light into voltage [76]. These metasurfaces achieve near-unity absorption (in particular, 97% at a wavelength of 532 nm) and room-temperature operation, making them suitable for environmental sensing, detection, and ultra-wideband spectroscopy. Asymmetric metasurface designs allow polarization-selective detection, adding a further dimension of light control.

One notable breakthrough in this field involves hybrid silicon–aluminum nanostructures that bridge the traditional gap between color filters and sensors, achieving “zero distance” for submicrometer pixel sizes [77]. These nanostructures utilize hybrid Mie-plasmonic resonance to enable color-selective absorption, facilitating charge separation through a Schottky barrier at the silicon–aluminum interface. This configuration significantly minimizes optical crosstalk, allowing for ultra-high pixel densities and expanded functionalities, such as polarization sensitivity and ultraviolet detection. By adjusting the nanodisk dimensions, fine-tuned absorption peaks can be realized, presenting a compelling alternative to conventional dye-based filters in imaging applications. This advancement paves the way for next-generation image sensors that offer superior resolution and efficiency compared to traditional designs.

A polarization-sensitive plasmonic photodetector has emerged using aluminum nanoantennas integrated with silicon photodetectors. These nanoantennas selectively filter light based on polarization through localized plasmon resonances [78]. The device employs back-to-back Schottky photodetectors sensitive to orthogonal polarization states, generating a differential electrical signal for precise polarization detection. This design demonstrates broadband operation (specifically, high performance in the wavelength range of ∼500–800 nm) and robustness against intensity fluctuations, presenting a compact and CMOS-compatible solution for optical communications, imaging, and data storage.

An enhanced photoresponse can be achieved in organic photodetectors by taking advantage of metasurface technology to improve photodetector performance [79]. Using the generalized Snell’s law, the incorporation of a phase-gradient metasurface that redirects incident light at specific angles increases the optical path length within the thin active layer. This design leads to substantial enhancements in responsivity (ranging from 1.5 to 2 times) across a relatively broad wavelength range of 560–690 nm, offering a practical and efficient method for boosting organic photodetector performance without complex material modifications.

**Figure 7:**
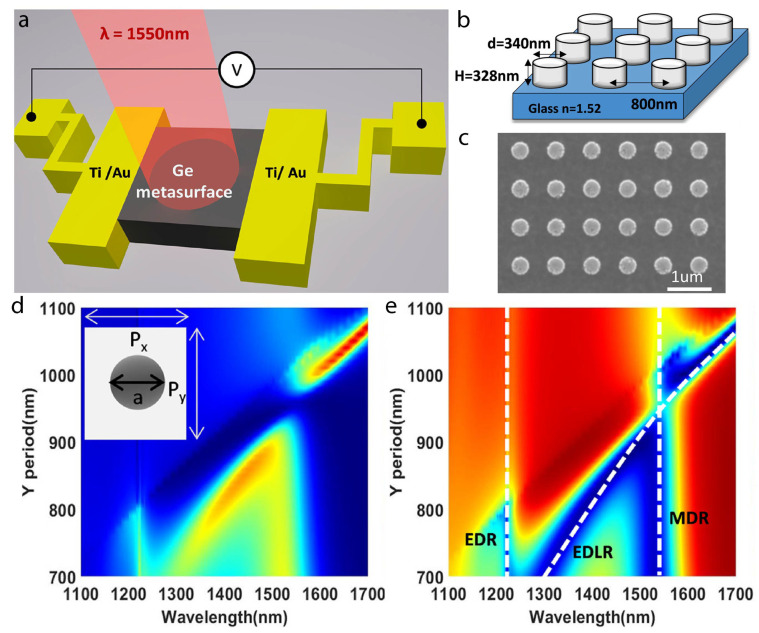
(**a**) Graphical design of a photodetector with a Ge metasurface with contact electrodes on the right and left sides of the device. (**b**) Schematic of Ge metasurface nanoantenna arrays on a glass substrate with a period of 800 nm. (**c**) Top-view scanning electron microscopy (SEM) image of Ge metasurface nanoantenna arrays. (**d**) Simulation results of the reflectance spectra for different transverse periods demonstrating the suppression of reflectance when the Kerker condition is satisfied. (**e**) Simulation of the transmittance spectra for different transverse periods, showing the overlap of the ED lattice resonance and MD resonance. (**a**–**e**) Reproduced with permission from [80]. Copyright 2022 by American Chemical Society.

Germanium metasurface photodetectors have been engineered for optical communication within the O and C bands, featuring a 2D periodic array of Ge nanoantennas (Figure 7). This innovative structure enhances light absorption by a factor of six compared to unpatterned Ge films, leveraging the resonant lattice Kerker effect that arises from the overlap of Mie and ED lattice resonances [80]. This resonant coupling produces strong near-field enhancement within the nanoantennas, leading to a six-fold increase in photocurrent. The Ge metasurface design thus holds significant promise for narrowband photodetection in the near-infrared spectrum, making it highly relevant for applications such as optical filtering, sensing, and telecommunications, where precision and efficiency are paramount.

Infrared sensors are essential elements in diverse cutting-edge applications that aim to detect and react to infrared radiation. These detectors function by identifying and transforming infrared waves into quantifiable electrical signals through materials and architectures that respond to wavelengths within the infrared domain. Traditional semiconductor compounds used in infrared sensors encompass indium antimonide InSb, indium arsenide InAs, and mercury cadmium telluride HgCdTe (or MCT). These substances are selected due to their appropriate energy bandgaps, enabling the efficient absorption of infrared photons within atmospheric transmission bands, notably the mid-wave infrared (MWIR: 3–5 μm) and long-wave infrared (LWIR: 8–12 μm) spectral regions. The detection principle generally involves the excitation of charge carriers from the valence band to the conduction band upon photon absorption, thereby producing an electrical response. A key characteristic of these sensors, narrowband detection, is related to their ability to selectively respond to a confined wavelength range, thereby enhancing precision and sensitivity in spectral acquisition. Unlike conventional materials, titanium oxides have gained attention as viable narrow-bandgap substitutes as a result of their environmentally benign nature. Titanium dioxide, TiO_2_, is recognized as nontoxic, abundantly available, cost-efficient, and eco-friendly. This compound has exhibited significant potential across various domains, including solar energy conversion, visible-light-driven photocatalysis, and biomedical applications. Although extensive studies have been conducted on the wide-bandgap rutile phase of TiO_2_, its 3.3 eV bandgap is excessively large for infrared applications. Recently, an advanced infrared sensor leveraging Ti_2_O_3_ has been reported, demonstrating enhanced performance through the incorporation of a resonant metasurface [81]. The results reveal that the absorptance of the ultra-thin Ti_2_O_3_ layer markedly improves, nearing complete absorption, because of the resonant metasurface, which facilitates efficient scattering within the active region. Studies confirm that the Ti_2_O_3_-based detector exhibits superior light absorption compared to conventional infrared materials such as InSb, InAs, and HgCdTe.

Recent advances in metasurface technology have significantly enhanced the capabilities of photodetectors across various spectral ranges, demonstrating promising applications in mid-IR detection, multidimensional light sensing, and high-speed thermal imaging. These innovations offer improved light–matter interaction control, increased efficiency, sensitivity, and compact integration, which are critical to the development of optoelectronic devices. Multipoles are crucial in enhancing the broadband absorption of mid-IR pyroelectric detectors. The contributions of various resonant modes, including ED, MD, and EQ resonances, collectively lead to optimized light absorption. ED resonance dominates the entire spectral range, particularly influencing absorption peaks at 3.94 μm and 4.8 μm. MD and EQ resonances contribute significantly at 3.94 μm, enhancing the overall absorption [82]. This has been achieved using interacting metasurface structural elements, such as nanogrids and nanopatches. The resonance behavior is further influenced by the dimensions of the nanostructures and the thickness of the dielectric layer, which allow the fine-tuning of the absorption peaks through adjustments in size and geometry. These multipolar interactions create strong localized electric fields and enable efficient photothermal energy conversion, leading to improved performance of the pyroelectric detector.

For mid-IR photodetection, a hybrid metal–dielectric optical antenna has been fabricated to enhance the response of Ge/Si quantum-dot infrared photodetectors [83]. This metasurface design effectively leverages surface plasmon resonance and Rayleigh anomalies, resulting in a remarkable 15-fold increase in peak responsivity at 4.4 μm. This hybrid configuration surpasses the performance of traditional plasmonic structures by achieving superior light coupling and optimized field distribution. Experimental validation at cryogenic temperatures confirms these performance enhancements, highlighting the potential of metasurface-based optimization for the advancement of mid-IR photodetection technologies.

A novel metasurface-integrated graphene photodetector has been utilized, offering a compact alternative to conventional bulky systems that are typically required to capture intensity, polarization, and wavelength information [84]. By integrating a metasurface with graphene, this device encodes vectorial photocurrents that vary based on the incident light properties. This integration allows for the simultaneous detection of polarization and wavelength across the 1–8 μm spectrum. These innovations highlight the transformative potential of metasurfaces in advancing photodetector technology and improving diverse applications.

Finally, advanced designs involving long-wavelength infrared detection can be mentioned. While the graphene-based detector excels in multidimensional light detection, the hybrid metasurface-coupled quantum-well infrared photodetectors and LEDs (QWIP-LEDs) demonstrate how metasurfaces can also revolutionize thermal imaging by significantly enhancing upconversion efficiency. Another significant breakthrough involves high-speed, pixel-less thermal imaging through hybrid metasurface-coupled QWIP-LEDs [85]. Traditional focal plane arrays often experience thermal mismatch issues, limiting performance. However, upconversion pixel-less imaging addresses these challenges by converting long-wave infrared signals into near-infrared signals. Despite this advancement, low upconversion efficiency has been a bottleneck for real-time imaging applications. The introduction of a hybrid metasurface structure enhances the light-coupling efficiency and the-light extraction efficiency in the QWIP-LED. It achieves a 10-fold increase in upconversion efficiency, and this improvement enables a theoretical integration time of just 3.3 ms and supports frame rates exceeding 300 Hz. These advancements position this technology as highly viable for defense, biomedical imaging, and advanced sensing applications.

Advancements in QWIPs have focused on increasing performance through innovative metasurface designs. A notable development involves the integration of a double L-shaped chiral metasurface with GaAs/AlGaAs QWIPs, achieving a circular polarization extinction ratio of 45 within the 7–9 μm range, surpassing earlier single L-shaped configurations [86]. Operating in microcavity, surface plasmon polariton, and hybrid modes, the device enhances absorption efficiency and achieves a peak coupling efficiency of 2700% at 7.9 μm. The double-L-shaped metasurface differentiates between left- and right-handed circularly polarized light, enabling high polarization selectivity even at large incident angles. This architecture offers significant potential for polarization-sensitive detection and spectral imaging applications.

Metasurface-enhanced photodetectors have emerged as a groundbreaking advancement, revolutionizing the capabilities of modern optoelectronic devices. By offering unprecedented control over light–matter interactions, these technologies have unlocked new potential in applications ranging from high-speed thermal imaging to ultra-broadband photodetection and multidimensional light sensing. Innovations such as hybrid silicon–aluminum nanostructures, Ge nanoantenna arrays, and MoS_2_-based plasmonic metasurfaces have significantly improved responsivity, detection precision, and spectral selectivity. Metasurface-based photodetectors are set to revolutionize optoelectronic technologies by offering enhanced control over light–matter interactions, increasing efficiency, sensitivity, and versatility.

Photogating is a phenomenon in which incident light modifies the charge carrier density in a material, leading to a persistent change in conductivity even after illumination is removed. This effect arises from trapped photogenerated carriers that create an internal electric field, enhancing the photocurrent and extending carrier lifetimes in photodetectors. Recent studies have demonstrated that photogating effects in metasurface-enhanced photodetectors can be significantly influenced by material composition, device architecture, and excitation conditions. Photogating effects in metasurfaces have emerged as a pivotal mechanism for enhancing photodetector performance, particularly in devices that integrate two-dimensional materials, such as transition metal dichalcogenides. Hybrid structures incorporating transition metal dichalcogenides with plasmonic or dielectric metasurfaces have shown strong photogating-induced photocurrent enhancements due to prolonged carrier lifetimes and localized field amplification. In these systems, incident light generates electron–hole pairs, with some carriers becoming trapped at defect sites or interfaces, leading to a persistent internal electric field that modulates the conductivity of the material. This effect can be significantly amplified by incorporating plasmonic metasurfaces, which localize and enhance electromagnetic fields at the nanoscale. Research on MoS_2_-based photodetectors integrated with plasmonic hafnium nitride metasurfaces reported an extraordinary 118-fold increase in photocurrent, attributed to enhanced light confinement and charge accumulation effects [73]. Integrating MoS_2_ with nitride-based resonant plasmonic metasurfaces achieved a detectivity of 2.58 × 10^12^ Jones, attributed to the plasmon-enhanced photogating effect [73]. Similarly, the concept of a dual-photogating effect at the MoS_2_/substrate interface has been proposed, resulting in ultra-high photoresponsivity and on/off ratios [87]. These advances show the potential for engineering photogating effects through metasurface integration to develop highly sensitive and efficient photodetectors. In general, while plasmonic metasurfaces provide stronger field enhancement, dielectric metasurfaces can offer lower losses and greater spectral tunability, emphasizing the importance of material selection for optimizing photodetector performance.

Table 1 presents a comparison of metasurface-based photodetectors, highlighting variations in design and their corresponding impact on performance metrics such as responsivity and detection efficiency. The table also indicates the active materials used in each device, emphasizing their role in tuning optical and electronic properties. Furthermore, the operating wavelength range is highlighted, illustrating how different material choices and structural configurations enable detection across various spectral regions.

## 5. Bound States in the Continuum

In this section of the review, we provide a comprehensive discussion of BICs within periodic photonic structures and metasurface, highlighting their unique characteristics. Bound states in the continuum (BICs) have emerged as a pivotal concept in modern photonics. They represent a class of states that are localized within a continuum of radiating modes but do not radiate energy. This phenomenon challenges conventional wave theory, which predicts that states within the continuum should inevitably couple to radiation and dissipate energy. Instead, BICs maintain their localization through specific interference effects and symmetry properties, which offer significant implications for the design of advanced optical devices and systems [88,89,90,91,92,93]. Although the theoretical foundation of BICs can be traced back to quantum mechanics, where von Neumann and Wigner first identified such states in 1929, their applications have since expanded into various domains, including nanophotonics, acoustics, and optomechanics [94,95,96,97,98,99,100]. Friedrich and Wintgen, in 1985, observed a BIC in a two-resonator system by fine-tuning the system’s parameters, resulting in destructive interference of the two resonances, leading to the vanishing of the linewidth in one of the resonances. This BIC is referred to as an accidental or parametric BIC because this can occur at any point in the Brillouin zone off-Γ-point.

The concept of BICs was first introduced in the field of photonics by Shabanov and colleagues in 2008, which laid the theoretical understanding of BICs in photonic structures [101,102,103,104,105]. Subsequently, an experimental realization with a configuration of interconnected optical waveguides was achieved by Plotnik and colleagues in 2011, and they demonstrated that an antisymmetric mode could propagate without any loss to the continuum [106]. This type of observed BIC is termed symmetry-protected (SP) BIC because of the requirement that all antisymmetric modes decouple at the Γ-point, one of the most highly symmetric points in the Brillouin zone. As a result, SP-BICs exhibit an infinite quality factor (*Q*-factor), meaning they can remain localized without radiative losses, making them ideal for applications requiring high sensitivity and precision, such as optical sensing [107].

Hsu et al. made a significant contribution to the field of BICs in 2013 by demonstrating the existence of embedded photonic bound states in a single layer of a free-standing silicon nitride Si_3_N_4_ thin film forming photonic crystals [108]. The distinction between these two types of bound states is crucial for the design of photonic devices. SP-BICs are often employed for their high *Q*-factors and stability, making them suitable for applications in sensors and resonators [109]. On the other hand, accidental BICs, while potentially less stable, can be engineered through careful designs to achieve specific functionalities, such as enhanced light–matter interactions or tunable resonances [110]. Thus, since the theoretical realization of BICs in the field of optics in 2008 and the subsequent experimental demonstration in 2011 and 2013, there have been significant advancements in understanding and utilizing these phenomena in various photonic and metasurface structures.

Zhen and colleagues explored the topological nature of BICs, showing that bound states can exhibit robust properties due to their topological characteristics [111]. This work opened new avenues for exploring the interplay between topology and photonic states, leading to innovative designs in photonic devices. The years following 2016 saw a surge in research focused on the applications of BICs in photonic structures and systems. Liang and colleagues provided insight into anisotropic plasmonic metasurfaces, demonstrating that BICs can be achieved even in materials traditionally associated with high losses, thus broadening the scope of potential applications in nanophotonics [112].

The effects of structural disorder on BICs in periodic structures were investigated by Gao and colleagues in 2019, and they demonstrated that BICs can still exist under certain disordered conditions [113]. These findings are crucial for real-world applications where imperfections are inevitable. Furthermore, Xu and Shi focused on the integration of high-quality metagratings and silicon waveguides to support BICs, showing the versatility of these states applied on various photonic platforms [114]. Moreover, the concept of super quasi-BICs has been introduced, where enhancements in quality factors are achieved through the merging of multiple BICs, highlighting the ongoing innovation of these states [115,116]. Recent studies have continued to push the boundaries of BIC research. The concept of optical Moiré BIC was introduced by Qin and colleagues in 2024, accompanied by a demonstration of how Moiré patterns can be engineered to create high-*Q* photonic integrated structures [117].

Promising developments in nonradiating photonics and BIC include investigations of resonant nanostructures [118,119], acousto–optic modulation of photonic BICs [120], efficient higher harmonic generation by harnessing bound states [121], BICs in an integrated Gires–Tournois interferometer [122], and guiding light through optical BICs for ultra-high-*Q*-factor microresonators [123]. Other effects involving the formation of bound states have been explored, with a particular impact of optical bistability with BIC in dielectric gratings [124], BIC Rabi oscillations [125], defect-induced BICs [126], enhancing strong coupling of transition metal dichalcogenide monolayers by BIC [127], BICs in waveguide arrays [128], SP BICs in graphene nanoribbons [129], SP BICs for integrated photonics [130], and critical field enhancement of asymptotic optical BICs [131], to name just a few.

In periodic photonic structures, BICs are generally classified into three categories based on their distinct characteristics [132]. In these structures, BICs can be observed as a result of tuning structural parameters such as the geometry and refractive index of the materials involved. This tuning allows for precise phase-matching conditions necessary for destructive interference to occur, thereby enabling the formation of these bound states. These types of BIC are called Fredrich–Wintgen (FW) BICs, and they occur at off-Γ-points in the momentum space.

**Figure 8:**
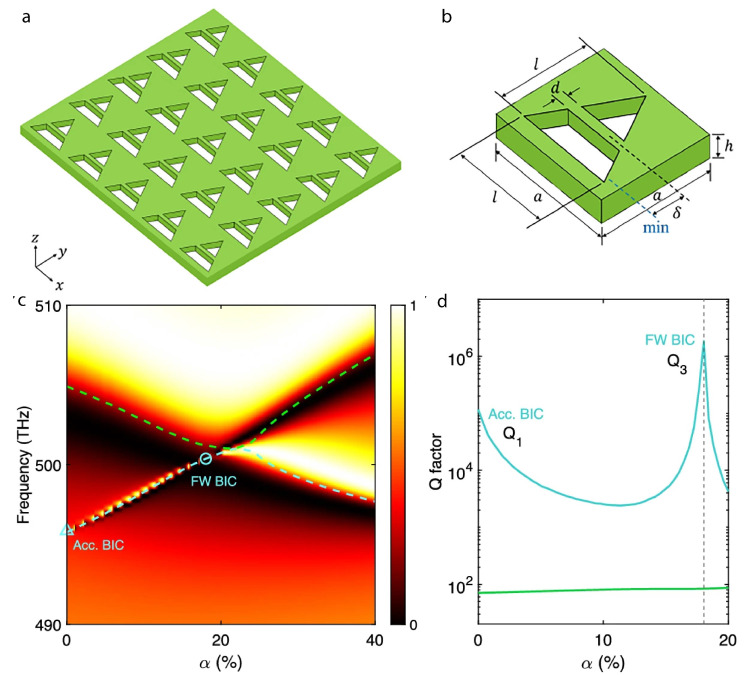
(**a**,**b**) Schematic representation of the divided triangular holes illustrating the square lattice (panel **a**)) and unit cell (panel **b**)). (**c**) FW BIC emerging in the transmittance diagram as a function of the height ratio parameter α and the frequency of the two-part divided triangular hole metasurface. (**d**) *Q*-factors of the dispersion bands associated with the metasurface structure. (**a**–**d**) Reproduced with permission from [133]. Copyright 2024 by Springer Nature.

**Figure 9:**
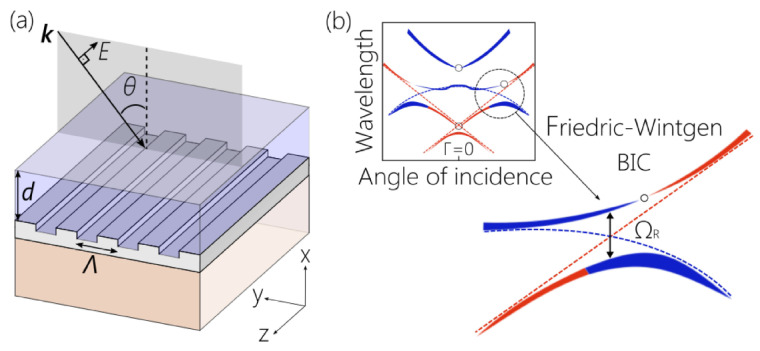
Formation of Friedrich–Wintgen (FW) and symmetry-protected (SP) BICs within a one-dimensional hybrid plasmonic-photonic structure. (**a**) Schematics of the grating. (**b**) Band diagram. The band structure highlights the off-Γ FW BIC at the avoided crossing and the Γ-point SP BIC. (**a**,**b**) Reproduced with permission from [102]. Copyright 2018 by American Physical Society.

The formation of FW BICs within a novel class of photonic structures was investigated by Chern and Hsu in 2024 (Figure 8) and became known as divided triangular hole metasurfaces [133]. The authors attributed the formation of the FW BICs to the interference of resonant modes within the metasurface, which occurs when two or more resonances approach each other in frequency space. Specifically, the divided triangular hole configuration allows the tuning of structural parameters, enabling the precise phase matching necessary for destructive interference. The authors highlight that these BICs are characterized by their non-radiative nature, as they cannot couple to far-field radiation, because their field symmetry is incompatible with available radiative channels. This characteristic leads to a high *Q*-factor reaching $10^{6}$, indicating that the modes are effectively trapped within the structure. Another example of FW BIC is in the one-dimensional plasmonic grating, shown in Figure 9, where FW BICs appear near the avoided crossings of two bands in the band structure [102]. In this case, the photonic waveguide modes are strongly coupled to the gap plasmons in the grating, leading to spectral features while avoiding crossing

**Figure 10:**
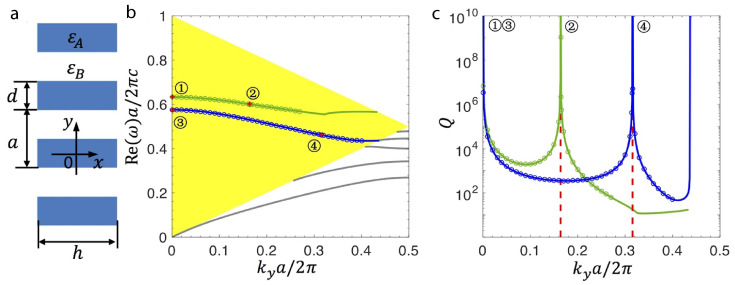
(**a**) Schematic representation of a one-dimensional photonic crystal slab embedded in a homogeneous dielectric medium. (**b**) Band structure corresponding to the structure. The region highlighted in yellow denotes where a single leaky channel exists in the surrounding space. The light blue (green) continuous line represents the dispersion of guided resonances that are symmetric (antisymmetric) with respect to the *x*-direction. Red plus symbols show BIC points. Marks ➀ and ➂ emerge because of the symmetry protection, whereas ➁ and ➃ do not. The circular marks correspond to the results of the full-wave numerical simulations. The gray lines depict guided modes emerging below the light line. (**c**) Quality factors of the guided resonances and BICs. The SP BICs are denoted as ➀ and ➂, while the accidental BICs are indicated as ➁ and ➃. (**a**–**c**) Reproduced with permission from [94]. Copyright 2016 by Springer Nature.

A second type of BIC, commonly referred to as SP BIC, is located at the center of the momentum space, specifically at the Γ-point. These states arise from the intrinsic symmetries present within the structure, where a mode cannot couple to far-field radiation because its field symmetry is incompatible with the available radiative channels. This type of BIC has been observed in various photonic structures and designs. For example, the configurations illustrated in Figure 9 and Figure 10 support SP BICs.

In Figure 9, the SP BIC at the Γ-point originates from the symmetry of the plasmonic modes, while the SP photonic BIC arises from the symmetry of the photonic modes [102]. The one-dimensional photonic-crystal-slab structure is depicted in Figure 10a, and it supports an SP BIC depicted in panel (b) as (➀ and ➂), which can be found at the Γ-point ($k_{y}=0$) [94]. In this case, the radiation vanishes because $E_{z}$ is odd with respect to the *y*-direction for the resonance but even with respect to the *y*-direction for the radiating wave, as illustrated in Figure 10b.

The third type of BIC, sometimes referred to as FW BIC, is known as an accidental BIC. This type of behavior occurs as a result of the tuning of the structural parameters. Consequently, accidental BICs evolve in the momentum space with changes in these parameters. They manifest at off-Γ-points due to destructive interference of the resonances under the correct phase-matching conditions. The structure shown in Figure 10a supports an accidental BIC with a non-zero Bloch vector represented in Figure 10b as ➁ and ➃. The quality factor shown in Figure 10c diverges at the BIC points, where accidental BICs occur at $k_{y}=0.3156$ (2π/*a*) for even-in-*x*-modes and at $k_{y}=0.1640$ (2π/*a*) for odd-in-*x*-modes. The achieved *Q*-factor for the accidental BIC reaches $10^{10}$ at $k_{y}=0.1640$, indicated as ➁.

The conception of a stratified mixed plasmonic–dielectric metasurface that allows robust mode interaction and the emergence of bound states in the continuum has been documented, yielding resonances with an elevated quality factor [134]. The ability to manipulate Fano resonances and adjust Rabi splitting has been exhibited through variations in the nanoantenna dimensions. The extended Kerker phenomenon in a dual-patterned arrangement of silicon nanodisks, which permits the modulation of collective modes and introduces novel photonic functionalities alongside enhanced sensing performance, has likewise been empirically validated. These results have significant potential for the advancement of plasmonic sensors that exploit intense light–matter coupling in composite metasurfaces.

The principles of BICs enable the conception and realization of optical resonant configurations with a high quality factor by utilizing dielectric frameworks with minimal dissipation. Nevertheless, BIC represents a universal wave phenomenon that should manifest itself in various systems, including metal–dielectric structures sustaining surface plasmon polaritons, where optical resonances are constrained by absorption. A comprehensive approach to attaining high-*Q* resonances in plasmonic metasurfaces has been formulated by proficiently adjusting the resonant modes from localized to nonlocal regimes, thereby shifting from quasi-isolated confined resonances to extended resonant states characterized by strong coupling between adjacent structural meta-units [135].

Multipolar resonances and BICs are deeply intertwined in the design of metasurfaces, offering unique opportunities to tailor light–matter interactions at the nanoscale. Multipolar resonances arise from the spatial distribution of electric and magnetic fields in structured nanoantennas, enabling highly directional scattering and the enhancement of absorption and light–matter interaction. BICs, on the other hand, are special optical modes that remain completely localized within a metastructure despite existing within the continuum of radiative states. This suppression of radiation losses is achieved through symmetry protection or parameter tuning, leading to extremely high-quality factors. When multipolar modes interfere constructively, they can induce quasi-BIC states, where a small perturbation allows controlled radiation leakage. This interplay provides a mechanism for engineering resonances with customizable linewidths, enabling enhanced field confinement, strong nonlinear responses, and precise spectral control in metasurface-based devices.

The practical applications of multipolar resonances and BICs span a wide range of photonic technologies, particularly in sensing, imaging, and energy harvesting. In photodetectors, metasurfaces leveraging multipolar resonances can enhance light absorption by selectively tuning electromagnetic responses, improving quantum efficiency and spectral selectivity [73,80]. The near-field enhancement associated with BICs further amplifies these effects, making them valuable for applications such as infrared detection, Raman spectroscopy, and nonlinear optics. Furthermore, BICs have been employed in lasing applications, where their high-*Q* resonances enable low-threshold lasing with strong directional emission. Similarly, in optical computing and communications, the ability to engineer high-*Q* resonances using multipolar modes allows for ultra-narrowband filters and modulators. By harnessing these fundamental phenomena, metasurface-based technologies continue to push the boundaries of miniaturized, high-performance photonic devices.

## 6. Conclusions

Multipolar resonances, BIC, and tailored light–matter interactions provide a robust framework for the development of metasurface-based photodetectors. The control of multipolar modes enables the precise manipulation of scattering and absorption, while BICs facilitate high-Q confinement, enhancing the optical energy concentration. These effects significantly impact spontaneous emission, enabling improved quantum efficiency and photon extraction. By integrating these principles, metasurfaces can be engineered to achieve superior photodetection performance, offering enhanced sensitivity, spectral selectivity, and ultra-compact device architectures. Future research will further optimize these platforms for applications in imaging, sensing, and quantum photonics.

## Figures and Tables

**Table 1:** Metasurface-based photodetectors vary in design, which in turn influences their performance. Active material and operating wavelength are also discussed.

Metasurface-Based Photodetector Design	Active Element	Operating Wavelengths	Key Performance	Ref.
Resonant coupling of plasmonic metasurface with monolayer single-crystal MoS_2_	MoS_2_	650–670 nm	118-fold photocurrent enhancement, high detectivity of 2.58 × 10^12^ Jones, a record-low dark current of 8 pA, and possibility of wafer-scale integration	[73]
Monolayer MoS_2_ and thin insulator sandwiched between metal electrode nanostripe array and metal substrate operating based on gap surface plasmons	MoS_2_	650 nm	Fast response and 30-fold polarization sensitivity	[74]
Phase-change material Sb_2_S_3_ nanograting with Fabry–Perot resonances on top of a continuous meta layer	Sb_2_S_3_	475–650 nm	Photocurrent for the TM amos. state can reach 0.60μAm^−2^ around the wavelength of 570 nm, while the photocurrent response for TM crys. state is 0.12 μAm^−2^ at 670 nm	[75]
Sb_2_Te_3_ metasurface consisting of nanoantennas with Mie resonances (including ED and MD) etched into the Sb_2_Te_3_ film with Fabry-Pérot resonances	Sb_2_Te_3_	700 nm to 14 μm	Ultra-wideband thermoelectric detection operating at room temperature, linear polarization sensitivity, and maximum absorptance of ≈97% at the wavelength 532 nm	[76]
Hybrid plasmonic-Mie resonances (including MD) in silicon-aluminum nanostructures	p-Si	450–650 nm	Color sensitivity achieved with strong size-dependent absorption peaks, the extreme limit of zero distance between color filters and sensors, the responsivity of the nanostructured detector element is 47.2 mA/W, and external quantum efficiency of 9%	[77]
Metal–semiconductor –metal photodiode with tailored arrays of plasmonic aluminum nanoantennas (rectangular or chiral pattern)	n-Si	500–800 nm	Differentiation of optical polarization in broadband range, selective screening of the underlying active region from light with a particular polarization state, and full CMOS compatibility	[78]
Metasurface-enhanced photoresponse in organic photodiode	P3HT:PC_71_ BM	560–690 nm	Metasurface engineered to deflect the beam inside the organic material into the desired angle and responsivity enhanced by 1.5× and 2× at the wavelength of 560 nm and 690 nm	[79]
Periodic nanoantenna array with ED and MD resonances and lattice Kerker effect	Ge	1.4–1.6 μm, max @1.55 μm	6-fold absorption increase in the near-infrared range	[80]
Collective Mie resonances in silicon nanocuboids enhancing scattering in the active layer underneath the metasurface	Ti_2_O_3_	3–5 μm	With a thin resonant metasurface, absorption in active Ti_2_O_3_ layer is predicted to significantly improved up to >80%	[81]
Pyroelectric nanogrid-patch units with Fabry–Perot cavity, ED, MD, and EQ resonances facilitated by metal-insulator-metal layout	LiTaO_3_	3–5 μm	Average absorption of 94.2% in the mid-infrared range, noncryogenic, and thermal response enhanced ×2.6	[82]
Hybrid metal–dielectric metasurface with silicon pillar array protruding through holes in metal film and enhanced by surface plasmon resonance at the Rayleigh anomaly	Ge/Si QDs	2–6 μm, max @4.4 μm	4× photoresponse enhancement compared to a standard hole array plasmonic design, and 15× increase in peak responsivity at 4.4 μm compared to a bare quantum dot infrared photodetectors	[83]
Integrated asymmetric dual-arm plasmonic nanostructures on top of graphene flakes	Graphene flakes	1–8 μm	Wavelength prediction accuracy of 0.5μm over the 1–8μm range of multi-dimensional information (intensity, polarization, and spectrum) and ability to differentiate between left and right circularly polarized light	[84]
Upconversion pixel-less thermal imaging with grating metasurface-coupled QWIP-LED	GaAs/AlGaAs QWs	9 μm	Theoretical prediction of high optical coupling efficiency of 7.7, upconversion efficiency of 3.6, the light extraction efficiency of 4.4, and potential for frame rate >300 Hz (integration time 3.3 ms)	[85]
Metal-semiconductor-metal stack with chiral metasurface	GaAs/AlGaAs QWs	7–9 μm	Theoretical prediction of high circular polarization extinction ratio of 45, peak absorption of 0.8, and coupling efficiency of 2700% at 7.9 μm for left-handed circularly polarized light	[86]

## Data Availability

The data that support the findings of this study are available from the corresponding author upon reasonable request.

## Abbreviations

The following abbreviations are used in this manuscript:

BIC	Bound States in the Continuum
CMOS	Complementary Metal-Oxide-Semiconductor
ED	Electric Dipole
EOC	Electric Octupole
EQ	Electric Quadrupole
FW	Friedrich-Wintgen
GMR	Guided Mode Resonance
LDOS	Local Density of Optical States
LED	Light-Emitting Diode
Mid-IR	Mid-Infrared
MD	Magnetic Dipole
ML	Machine Learning
MMP	Multiple Multipole
MOC	Magnetic Octupole
MQ	Magnetic Quadrupole
MTD	Magnetic Toroidal Dipole
MRI	Moderate Refractive Index
PF	Purcell Factor
QD	Quantum Dot
QWIP	Quantum Well Infrared Photodetector
SEM	Scanning Electron Microscopy
SHG	Second-Harmonic Generation
SP	Symmetry Protected
SPP	Surface Plasmon Polariton
TD	Toroidal Dipole
TED	Total Electric Dipole
